# Influence of the number and timing of malaria episodes during pregnancy on prematurity and small-for-gestational-age in an area of low transmission

**DOI:** 10.1186/s12916-017-0877-6

**Published:** 2017-06-21

**Authors:** Kerryn A. Moore, Julie A. Simpson, Jacher Wiladphaingern, Aung Myat Min, Mupawjay Pimanpanarak, Moo Kho Paw, Jathee Raksuansak, Sasithon Pukrittayakamee, Freya J. I. Fowkes, Nicholas J. White, François Nosten, Rose McGready

**Affiliations:** 1 Centre for Epidemiology and Biostatistics, Melbourne School of Population and Global Health, The University of Melbourne, Melbourne, VIC Australia; 2 Macfarlane Burnet Institute for Medical Research and Public Health, Melbourne, VIC Australia; 3 Shoklo Malaria Research Unit, Mahidol-Oxford Tropical Medicine Research Unit, Faculty of Tropical Medicine, Mahidol University, Mae Sot, Thailand; 4 Mahidol-Oxford Tropical Medicine Research Unit, Faculty of Tropical Medicine, Mahidol University, Bangkok, Thailand; 5 Department of Epidemiology and Preventive Medicine and Department of Infectious Diseases, Monash University, Melbourne, VIC Australia; 6 Centre for Tropical Medicine and Global Health, Nuffield Department of Medicine, University of Oxford, Oxford, UK

**Keywords:** Malaria in pregnancy, Preterm birth, Small-for-gestational-age, Timing, Gestation

## Abstract

**Background:**

Most evidence on the association between malaria in pregnancy and adverse pregnancy outcomes focuses on falciparum malaria detected at birth. We assessed the association between the number and timing of falciparum and vivax malaria episodes during pregnancy on small-for-gestational-age (SGA) and preterm birth.

**Methods:**

We analysed observational data collected from antenatal clinics on the Thailand-Myanmar border (1986–2015). We assessed the effects of the total number of malaria episodes in pregnancy on SGA and the effects of malaria in pregnancy on SGA, very preterm birth, and late preterm birth, by the gestational age at malaria detection and treatment using logistic regression models with time-dependent malaria variables (monthly intervals). World Health Organisation definitions of very preterm birth (≥28 and <32 weeks) and late preterm birth (≥32 and <37 weeks) and international SGA standards were used.

**Results:**

Of 50,060 pregnant women followed, 8221 (16%) had malaria during their pregnancy. Of the 50,060 newborns, 10,005 (21%) were SGA, 540 (1%) were very preterm, and 4331 (9%) were late preterm. The rates of falciparum and vivax malaria were highest at 6 and 5 weeks’ gestation, respectively. The odds of SGA increased linearly by 1.13-fold (95% confidence interval: 1.09, 1.17) and 1.27-fold (1.21, 1.33) per episode of falciparum and vivax malaria, respectively. Falciparum malaria at any gestation period after 12–16 weeks and vivax malaria after 20–24 weeks were associated with SGA (falciparum odds ratio, OR range: 1.15–1.63 [*p* range: <0.001–0.094]; vivax OR range: 1.12–1.54 [*p* range: <0.001–0.138]). Falciparum malaria at any gestation period after 24–28 weeks was associated with either very or late preterm birth (OR range: 1.44–2.53; *p* range: <0.001–0.001). Vivax malaria at 24–28 weeks was associated with very preterm birth (OR: 1.79 [1.11, 2.90]), and vivax malaria at 28–32 weeks was associated with late preterm birth (OR: 1.23 [1.01, 1.50]). Many of these associations held for asymptomatic malaria.

**Conclusions:**

Protection against malaria should be started as early as possible in pregnancy. Malaria control and elimination efforts in the general population can avert the adverse consequences associated with treated asymptomatic malaria in pregnancy.

**Electronic supplementary material:**

The online version of this article (doi:10.1186/s12916-017-0877-6) contains supplementary material, which is available to authorized users.

## Background

Pregnant women are more susceptible to malaria, and they are more severely affected by it [1]. An estimated 125 million women are at risk of malaria in pregnancy every year [2]. Malaria in pregnancy is associated with adverse birth outcomes including intrauterine growth restriction (IUGR) and preterm birth [3–8]. Small-for-gestational-age (SGA), a proxy for IUGR, and preterm birth are strong predictors of infant mortality and morbidity, including disability, stunting, and non-communicable diseases in later life [9, 10]. The majority of published evidence on the adverse birth outcomes (principally low birthweight [LBW], a proxy measure for IUGR and/or preterm birth) associated with malaria in pregnancy have only investigated the impact of falciparum malaria detected at delivery, rather than malaria (including other species) during pregnancy.

There are few prospective studies assessing the effects of malaria during pregnancy in relation to gestational age [8, 11–22], especially for vivax malaria [8, 20, 21], and these studies have reached conflicting conclusions (Additional file 1). Falciparum malaria detected at delivery, but not during pregnancy, has consistently been associated with preterm birth [12, 17], and falciparum malaria during pregnancy and at delivery has consistently been associated with fetal growth-related outcomes (LBW, SGA, and IUGR) [11, 13, 15–19, 22]. However, the gestational age at which falciparum malaria is most strongly associated with these adverse outcomes varies between studies. Few of these studies looked at falciparum malaria in early pregnancy [14–16], only one looked at the effect of falciparum malaria within gestation windows of less than 3 months [18], and only one was conducted outside of Africa [21]. Several, but not all [13] studies have also shown a cumulative association between the number of falciparum malaria episodes detected and fetal growth-related outcomes (Additional file 1) [11, 14, 15, 22–25]. There have been no studies on the effect of the number of vivax malaria episodes in pregnancy and fetal growth-related outcomes or of falciparum malaria outside of Africa where transmission is low and falciparum and vivax malaria coexist.

Understanding how the consequences of malaria in pregnancy differ by the number of episodes and the gestational age at detection and treatment is important for the design and timing of interventions to prevent or control malaria in pregnancy. Here, in refugee and migrant women attending antenatal clinics (ANCs) on the Thailand-Myanmar border, we assess the rates of both vivax and falciparum malaria throughout pregnancy and also study how the number of episodes and the gestational age at detection and treatment affect the risk of SGA and preterm birth.

## Methods

### Study area and population

Since 1986, the Shoklo Malaria Research Unit (SMRU) has collected data on prospectively followed pregnant women attending ANCs on the Thailand-Myanmar border, including confirmed *Plasmodium* spp. infections and pregnancy outcomes. Médecins Sans Frontières contributed ANC data in the first 9 years. The Oxford Tropical Research Ethics Committee granted ethical approval for analysis of anonymised SMRU clinical records (OXTREC 28-09), and the Tak Community Advisory Board granted local ethical approval (TCAB-4/1/2015).

### Procedures

Women were encouraged to attend antenatal care early and return weekly throughout their pregnancy for malaria screening (finger-prick blood sample examined by trained microscopists) because there were no suitable preventive interventions for malaria in this region [25, 26]. Syphilis and HIV were not routinely tested for, but prevalence is very low [27]. When malaria parasites were detected, information on species, symptoms, fetal viability, and gestational age were recorded. Malaria was defined as the presence of asexual parasites in the peripheral blood and was counted per 500 leucocytes or 1000 erythrocytes. Women were also asked about recent antimalarial treatments administered at other clinics, and information on these malaria episodes was recorded retrospectively. Genotyping data were not available to classify recurrent episodes, which could be either novel, recrudescent, or a relapse in the case of vivax malaria [28].

Symptomatic malaria was defined as parasitaemia plus a temperature ≥37.5 °C or a history of fever in the past 48 hours. Vivax malaria was treated with chloroquine. Falciparum malaria was treated with quinine in all trimesters until 1995, and thereafter with quinine (plus clindamycin from 2007) in the first trimester and artemisinin-based treatments in the second and third trimesters (and in the first trimester for cases of severe malaria or hyperparasitaemia [>4% infected red blood cells]). Around 13% of women with malaria in pregnancy attending SMRU ANCs were part of antimalarial treatment trials and are included in this analysis (Additional file 2). Women with malaria were encouraged to have supervised antimalarial treatment, whether or not they were enrolled in a trial, because of the high prevalence of drug resistance in this region. Haematocrit was measured fortnightly. Maternal anaemia (haematocrit <30%) was treated with ferrous sulphate and folic acid. Gestational age was predominantly estimated by fundal height measurement (1986–1994), the Dubowitz Gestational Age Assessment (1992–2002), and ultrasound biometry (2001 to the present) [29]. Birthweight was measured within 72 hours of delivery using electronic Seca medical scales with a precision of 10 g.

Primary exposures were the number of falciparum or vivax malaria episodes in pregnancy, and the gestational age of malaria detection and treatment. Primary outcomes were very preterm (birth at ≥28 and <32 weeks’ gestation), late preterm (birth ≥32 and <37 weeks’ gestation), and SGA (birthweight-for-gestational age below the INTERGROWTH-21st Project 10th centile) [30]. The majority of women deliver at SMRU delivery units with a skilled birth attendant. Pregnancy outcomes and details of the delivery are recorded for all pregnancies, including home and hospital deliveries. To assess the incidence rates of malaria over gestational age, we restricted our analysis to women who began antenatal screening before 10 weeks’ gestation. To assess the association between the number of malaria episodes and the gestational age at detection and birth outcomes, we included women attending SMRU clinics who delivered a singleton baby with an estimated gestational age, either live born or stillborn.

### Statistical analysis

First, we described the exposure — malaria in pregnancy — over gestational age. We used unadjusted Cox proportional hazards models to estimate the rates of vivax and falciparum malaria over gestational age; women were considered at risk of an initial malaria episode from their first antenatal consultation, and at risk of recurrent malaria from the gestational age of the initial episode. We plotted geometric mean parasitaemia and the proportion of malaria episodes that were symptomatic by the gestational age of malaria detection and treatment. The gestational age at malaria detection and treatment was assessed in monthly intervals (time *t*) until 28 weeks’ gestation (i.e. <4, ≥4 and <8, ≥8 and <12, ≥12 and <16, ≥16 and <20, ≥20 and <24, ≥24 and <28), followed by the intervals ≥28 and <32, ≥32 and < 37, and ≥37 weeks, which correspond to World Health Organisation definitions of very preterm birth, late preterm birth, and term delivery. Monthly intervals were chosen because they are specific but are not so small that the confidence intervals are unreasonable.

Next, to estimate the effect of malaria detection and treatment at time *t* and the total number of malaria episodes in pregnancy on very preterm birth, late preterm birth, and SGA, multivariable logistic regression modelling was performed with time-dependent malaria variables corresponding to the previously defined time intervals. Women without a positive screen within each interval were assumed to have no malaria within the respective interval. We did not perform the analysis of the effect of the total number of episodes on preterm birth because of feedback between the outcome (i.e. duration of pregnancy) and the exposure (total number of episodes). Linearity of the association between the number of malaria episodes and SGA was assessed using the likelihood ratio test, comparing models with the number of episodes fitted as a continuous or categorical variable; there was little evidence of non-linearity (falciparum: *p* = 0.819; vivax; *p* = 0.118). Models were adjusted for malaria history within the current pregnancy (i.e. malaria within each time interval), gravidity, clinic site, and yearly malaria incidence (Additional file 3). To differentiate between malaria species, women with both vivax and falciparum malaria (either mixed or sequential infections) in pregnancy were excluded. All analyses were performed in Stata Version 13 (StataCorp, College Station, TX, USA).

## Results

**Fig. 1:**
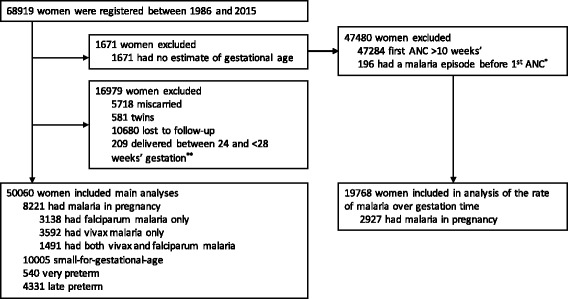
Study profile. ^*^These women had malaria episodes entered retrospectively, so were not undergoing antenatal screening from ≤10 weeks’ gestation. ^**^These newborns were live born, and are in a ‘grey zone’ between miscarriage and extreme preterm birth (birth at 24–28 weeks’ gestation) [53]

Between January 1986 and December 2015, 68,919 pregnant women presented to SMRU ANCs, of whom 50,060 (73%) were followed until delivery and gave birth to a singleton baby with an estimated gestational age (Fig. 1). Of these newborns, 10,005 (21%) were SGA, 540 (1%) were very preterm, and 4331 (9%) were late preterm. A total of 8221 women (16%) had malaria in pregnancy; these women were more likely to be younger, primigravid, to smoke, live in a migrant community rather than a refugee camp, and be anaemic at the last antenatal consultation (within 1 month of delivery for 90% of women) compared to women with no malaria in pregnancy (all *p* values <0.001) (Table 1). Antimalarial treatment was fully or partially supervised in 79% of women with malaria in pregnancy. Very preterm, late preterm, and SGA infants were more common in women who had malaria during their pregnancy (119 [2%], 870 [11%], and 2040 [27%], respectively) than in women without malaria in pregnancy (421 [1%], 3461 [8%], 7965 [20%], respectively).

**Table 1:** Cohort demographics

	Main analysis, *N* = 50,060		Analysis of rates of malaria over gestational age, *N* = 19,768	
Variable	No malaria, *N* = 41,839	Malaria, *N* = 8221	No malaria, *N* = 16,841	Malaria, *N* = 2927
EGA at 1st ANC, weeks’ gestation	15.6 {9.6, 23.8}, 0.0–42.0	14.8 {9.5, 22.1}, 0.0–41.1	7.6 {6.4, 8.7}, 0.0–10.0	7.5 {6.1, 8.7}, 0.0–10.0
EGA method				
Ultrasound	24,398 (58)	3809 (46)	12,385 (74)	1834 (63)
Dubowitz	9368 (22)	1727 (21)	1767 (10)	401 (14)
Fundal height	6171 (15)	2108 (26)	920 (5)	375 (13)
LMP	1902 (5)	577 (7)	1769 (11)	317 (11)
Maternal age, years	25 {21, 30}, 13–53	24 {20, 30}, 14–48	26 {21, 31}, 13–52	24 {20, 30}, 13–45
Primigravid	11,107 (27)	2383 (29)	4018 (24)	868 (30)
Anaemia^a^	3911 (11)	1369 (20)	1508 (9)	485 (17)
Haematocrit,^a^ %	34 {31, 36}, 10–53	33 {30, 35}, 16–49	34 {32, 37}, 10–49	33 {30, 35}, 16–48
Current smoker^b^	7551 (23)	1897 (35)	3653 (24)	817 (35)
Site				
Refugee	30,103 (72)	4649 (57)	12,218 (73)	1275 (44)
Migrant	11,736 (28)	3572 (43)	4623 (27)	1652 (56)
Place of delivery				
SMRU unit	24,502 (66)	3218 (46)	7612 (69)	977 (47)
At home	9406 (25)	3118 (45)	2509 (23)	878 (43)
Hospital	3149 (8)	598 (9)	984 (9)	205 (10)

Numbers are median {interquartile range}, range or frequency (%). Missing (main analysis): maternal age: 30 [<0.0%] (no malaria 25 [<0.0%]; malaria 5 [<0.0%]); smoking status 11,469 [23%] (no malaria 8651 [21%]; malaria 2818 [34%]); haematocrit 7012 [14%] (no malaria 5618 [13%]; malaria 1394 [17%])

*EGA* estimated gestational age, *ANC* antenatal clinic, *LMP* last menstrual period

^a^Measured at the last antenatal consultation (within 1 month of delivery for 90% of women)

^b^Smoking information was only routinely collected from 1997

Of the 8221 women with malaria, 3138 (38%) had falciparum malaria only, 3592 (44%) had vivax malaria only, and 1491 (18%) had both vivax and falciparum malaria (either sequential or mixed infections). Women with both vivax and falciparum malaria in pregnancy were excluded from subsequent analyses because of unknown interactions. Recurrent falciparum malaria (either novel or recrudescent) occurred in 860 women (27%): 602, 180, and 78 women had two, three, or four or more falciparum malaria episodes, respectively. Recurrent vivax malaria (either novel, recrudescent, or relapse) occurred in 1335 women (37%): 710, 322, and 303 women had two, three, or four or more vivax malaria episodes, respectively.

### Rates of falciparum and vivax malaria over gestational age

**Fig. 2:**
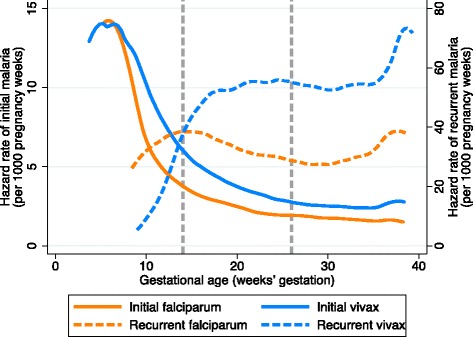
Rates of falciparum and vivax malaria over gestational age in women who started antenatal screening before 10 weeks’ gestation (*N* = 19,768). *Vertical dashed lines* are at 14 weeks’ and 28 weeks’ gestation, indicating the end of the first and second trimesters, respectively. Hazard rates were calculated per 1000 pregnancy weeks. For the hazard of initial malaria, women were censored at the gestational age of their first episode, birth, or time last seen. For recurrent malaria, women became at risk at the gestational age of their initial episode and were censored at the gestational age of their first recurrent episode, birth, or time last seen; we did not attempt to account for a post-treatment prophylactic effect because of the strong assumptions that this would require

In 19,768 women who began antenatal screening before 10 weeks’ gestation (Fig. 1 and Table 1), the rates of both initial falciparum and initial vivax malaria were highest in the first trimester and declined as the pregnancies progressed (Fig. 2). The rate of initial falciparum malaria was highest at 6.0 weeks’ gestation (14.2 episodes per 1000 pregnancy weeks), and the rate of initial vivax malaria peaked at 5.3 weeks’ gestation (14.0 episodes per 1000 pregnancy weeks) (Fig. 2). The rate of recurrence following an initial episode for falciparum malaria was highest on the cusp of the first and second trimesters (14.0 weeks’ gestation), declined slightly in the second trimester, and rose again during the third trimester (Fig. 2). The rate of recurrence following an initial episode for vivax malaria increased rapidly between the first and second trimesters, plateaued at around 20 weeks’ gestation, and then increased rapidly again near delivery (Fig. 2).

### Parasitaemia and symptoms by gestation at the time of detection

**Fig. 3:**
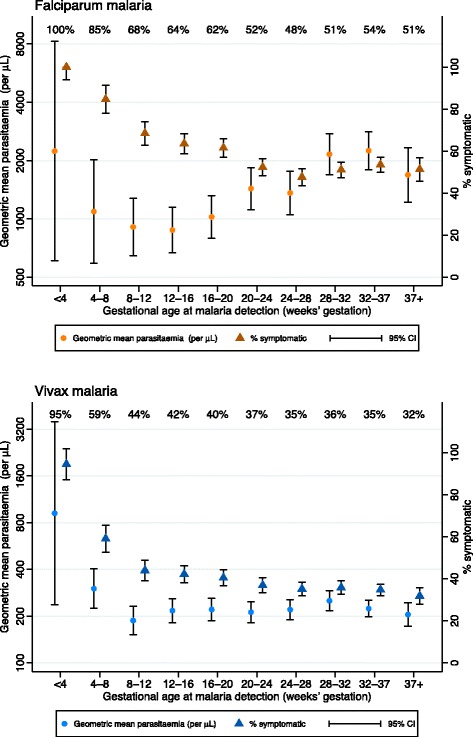
Descriptive statistics for parasitaemia and symptoms by the gestational age at malaria detection. *Orange*: falciparum malaria. *Blue*: vivax malaria. *CI* confidence interval

Parasitaemia was generally higher for falciparum malaria detected at <4 weeks’ gestation and after 20 weeks’ gestation than for falciparum malaria detected between 4 and 20 weeks’ gestation (Fig. 3). The proportion of falciparum malaria episodes that were symptomatic was high (above 60%) for episodes detected before 20 weeks’ gestation, especially relative to parasitaemia (Fig. 3). After 20 weeks’ gestation, around 50% of episodes detected were symptomatic, despite high parasitaemia relative to episodes detected before 20 weeks’ gestation (Fig. 3). Vivax parasitaemia was high for episodes detected very early in pregnancy (<8 weeks) but was relatively stable thereafter (Fig. 3). Similar to falciparum malaria, the proportion of vivax malaria episodes that were symptomatic declined over gestation, even after parasitaemia had stabilised (Fig. 3).

### Association between the number of malaria episodes in pregnancy and SGA

**Fig. 4:**
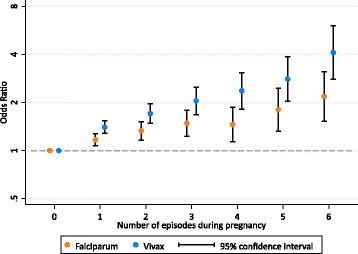
The association between the number of malaria episodes in pregnancy and small-for-gestational-age (*SGA*). Numbers are odds ratios with 95% confidence intervals. See Additional file 4 for a table version of this figure. The associations between the number of episodes and log_e_(odds) were linear for falciparum malaria (*p* = 0.819) and vivax malaria (*p* = 0.118). SGA was missing in 2636 [5%] (no malaria 2078 [5%]; malaria 558 [7%])

The odds of SGA increased linearly with the number of falciparum and vivax malaria episodes in pregnancy. The odds of SGA increased 1.13-fold (95% confidence interval, CI: 1.09, 1.17; *p* < 0.001) with each episode of falciparum malaria and 1.27-fold (95% CI: 1.21, 1.33; *p* < 0.001) for each episode of vivax malaria (Fig. 4). Associations were stronger for symptomatic malaria but were still present for asymptomatic malaria. The odds of SGA increased 1.17-fold (95% CI: 1.11, 1.24; *p* < 0.001) per symptomatic falciparum malaria episode and 1.10-fold (95% CI: 1.05, 1.15; *p* < 0.001) per asymptomatic falciparum malaria episode (Additional file 4). The odds of SGA increased 1.28-fold (95% CI: 1.21, 1.35; *p* < 0.001) per symptomatic vivax malaria episode and 1.24-fold (95% CI: 1.17, 1.31; *p* < 0.001) per asymptomatic vivax malaria episode (Additional file 4).

### Associations between the gestational age at malaria detection and SGA

**Fig. 5:**
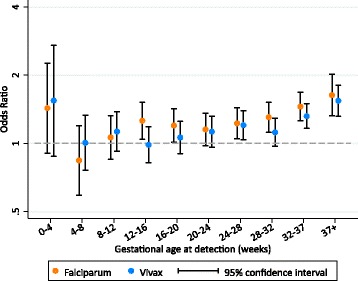
The association between the gestational age at falciparum or vivax malaria detection and treatment and small-for-gestational-age (SGA). See Additional file 5 for a table version of this figure, including differentiation between symptomatic and asymptomatic malaria. The reference group within each time interval is women with no malaria detected within each respective time interval. SGA was missing in 2636 [5%] (no malaria 2078 [5%]; malaria 558 [7%])

Falciparum malaria detected and treated at any gestational age after 12 weeks of pregnancy was associated with increased odds of SGA (odds ratio, OR range: 1.15–1.63; *p* value range: <0.001–0.094), especially after 28 weeks’ gestation (Fig. 5). There was no association between falciparum malaria detected and treated between 4 and 12 weeks’ gestation and SGA (OR range: 0.84–1.06 [*p* value range: 0.336–0.592]; pooled OR: 0.97 [95% CI: 0.80, 1.18; *p* = 0.764]) (Fig. 5). The magnitudes and patterns of association were similar for symptomatic and asymptomatic falciparum malaria (Additional file 5). Vivax malaria detected after 20 weeks’ gestation was also associated with increased odds of SGA (OR range: 1.12–1.54; *p* value range: <0.001–0.138), especially in the presence of symptoms, or after 32 weeks’ gestation, regardless of symptoms (Fig. 5; Additional file 5). There was no association between vivax malaria detected between 4 and 20 weeks’ gestation and SGA (OR range: 0.99–1.13 [*p* value range: 0.239–0.966]; pooled OR: 1.06 [95% CI; 0.94, 1.19; *p* = 0.380]) (Fig. 5), except after stratifying by symptoms (Additional file 5). The associations between both falciparum and vivax malaria detected before 4 weeks’ gestation were of notable magnitude but had very wide confidence intervals (1.43 [95% CI: 0.91, 2.26; *p* = 0.125; *N* with malaria: 90] and 1.54 [95% CI: 0.88, 2.72; *p* = 0.131; *N* with malaria: 89], respectively) (Fig. 5).

### Associations between the gestational age at malaria detection and preterm birth

**Fig. 6:**
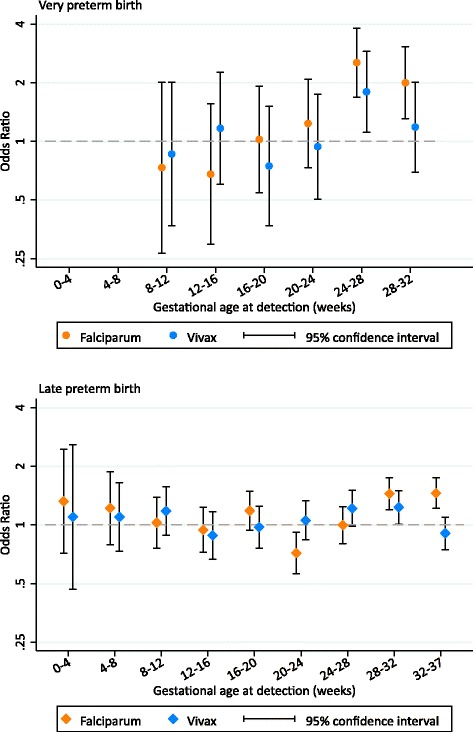
The association between the gestational age at falciparum or vivax malaria detection and treatment and preterm birth. The association between malaria at 0–4 and 4–8 weeks’ gestation and very preterm birth was not estimated due to zero events in the malaria groups. See Additional file 6 for a table version of this figure, including differentiation between symptomatic and asymptomatic malaria

Falciparum malaria was associated with birth between 28 and 32 weeks (i.e. very preterm birth) only if it was detected and treated after 24 weeks’ gestation. Falciparum malaria detected between 24 and 28 and between 28 and 32 weeks’ gestation increased the odds of very preterm birth 2.53-fold (95% CI: 1.69, 3.81; *p* < 0.001) and 2.00-fold (95% CI: 1.30, 3.06; *p* = 0.001), respectively (Fig. 6); the association at 24–28 weeks’ gestation was driven by both asymptomatic and symptomatic malaria, while the association at 28–32 weeks’ gestation was driven by symptomatic malaria (Additional file 6). Vivax malaria detected between 24 and 28 weeks’ gestation was also associated with very preterm birth (OR: 1.79; 95% CI: 1.11, 2.90; *p* = 0.017), but not between 28 and 32 weeks’ gestation (OR: 1.18; 95% CI: 0.69, 2.02; *p* = 0.536) (Fig. 6); these associations were similar for asymptomatic and symptomatic malaria (Additional file 6). There was no association between falciparum or vivax malaria detected and treated at earlier gestational ages and very preterm birth (falciparum OR range: 0.43–1.23 [*p* value range: 0.361–0.944]; vivax OR range: 0.75–1.17 [*p* value range: 0.418–0.844]; falciparum pooled OR: 0.97 [95% CI: 0.67, 1.40; *p* = 0.872]; vivax pooled OR: 0.82 [95% CI: 0.54, 1.25; *p* = 0.362]) (Fig. 6), regardless of symptoms (Additional file 6).

Falciparum malaria detected and treated between 28 and 32 and between 32 and 37 weeks’ gestation increased the odds of late preterm birth 1.44-fold (95% CI: 1.20, 1.75; *p* < 0.001) and 1.46-fold (95% CI: 1.22, 1.74; *p* < 0.001), respectively (Fig. 6). The association at 28–32 weeks’ gestation was driven by both asymptomatic and symptomatic malaria, while the association at 32–37 weeks’ gestation was driven by symptomatic malaria (Additional file 6). Surprisingly, falciparum malaria detected between 20 and 24 weeks’ gestation was associated with a reduction in the odds of late preterm birth (OR: 0.72; 95% CI: 0.56, 0.92; *p* = 0.008), but overall there was no association between falciparum malaria detected before 24 weeks’ gestation and late preterm birth (pooled OR: 0.96; 95% CI: 0.84, 1.11; *p* = 0.584) (Fig. 6), regardless of symptoms (Additional file 6). Vivax malaria detected between 28 and 32 weeks’ gestation increased the odds of late preterm birth 1.23-fold (95% CI: 1.01, 1.50; *p* = 0.039) (Fig. 6); this association was driven by symptomatic malaria (Additional file 6). There was also a borderline association between vivax malaria detected between 24 and 28 weeks’ gestation and late preterm birth (OR: 1.22; 95% CI: 0.98, 1.50; *p* = 0.072) (Fig. 6). There was no association between vivax malaria detected at earlier gestational ages and late preterm birth (OR range: 0.88–1.18 [*p* value range: 0.261–0.832]; pooled OR: 1.06 [95% CI: 0.92, 1.23; *p* = 0.424]) (Fig. 6), regardless of symptoms (Additional file 6).

## Discussion

This very large series of prospective observational data from antenatal clinics, with women presenting early in pregnancy and with weekly to fortnightly attendance, allows for the first time a detailed temporal characterisation of the association between falciparum and vivax malaria in pregnancy and SGA and preterm birth. It shows that the risks of both preterm birth and SGA vary depending on the gestational age at malaria detection and antimalarial treatment and on the total number of malaria episodes. The rate of malaria was highest during the first trimester. We have shown previously that first-trimester malaria is associated with an increased risk of miscarriage [31]; this series shows that if the fetus survives, there is no later increased risk of either preterm birth or SGA resulting from malaria treated in the first trimester. In contrast both falciparum and vivax malaria detected and treated from 12 weeks’ gestation were consistently associated with preterm birth and SGA. Many of the observed associations held in analyses of asymptomatic malaria specifically. The effect of falciparum and vivax malaria on SGA increased linearly with the number of episodes.

One inevitable limitation is that only the time of malaria detection (rather than actual timing of infection) and corresponding gestational age was known. Some women did not attend every week as recommended. But as most women attended weekly or fortnightly and blood smears were taken on every occasion, the inaccuracy in ascribing the timing of patent malaria infection is likely to be small. Inaccuracies in estimates of gestational age may have biased our results; however, we previously found that all estimation methods perform remarkably well in this population [29]. It should also be noted that these data were collected over a 30-year period, and the influence of changes over time in malaria transmission, methods used to estimate gestational age, antimalarials, and the quality of antenatal and obstetric care on our results is unknown, and is a key limitation. Although there are slight differences in results between sub-group analyses pre- and post-2001 (when ultrasound was introduced), these differences do not change our conclusions (Additional file 7). Genotyping data were not available to classify recurrent episodes, which could have been either novel, recrudescent, or a relapse in the case of vivax malaria [28]. We have also made many comparisons by assessing the association between malaria within monthly intervals, with differentiation by species and symptoms, and three adverse outcomes. We have addressed this by considering the magnitude of associations, their 95% confidence intervals and *p* values, and by focusing on trends in associations rather than on single associations within specific gestation windows. Reduced power to detect associations between malaria very early in pregnancy and adverse outcomes is another limitation.

Previous studies have found that the incidence or prevalence of falciparum malaria was highest in either the first or second trimester of pregnancy, but were unable to account for varying gestational ages at antenatal presentation and delivery, which is likely to differ between women with and without malaria [25, 32–35]. We found that the rate of falciparum and vivax malaria was highest in the first trimester. Because of the high prevalence of sub-clinical malaria in the general population [36], we postulate that the high rate of detection in the first trimester is largely an artefact of initiating active screening and treatment when women become pregnant, which would detect and clear infections that were probably present before conception. Even in this relatively low transmission setting, the rate of asymptomatic parasitaemia in adults commonly exceeds 5% even now, and was probably much higher when the programme began 30 years ago [36].

We found that falciparum malaria detected and treated from 12 weeks’ gestation onwards was associated with SGA, whether or not the mother was symptomatic. Others have also reported that falciparum malaria in early pregnancy (before 20 weeks’ gestation) was associated with intrauterine growth restriction or reduced birthweight [14, 19]. Additionally, it has been shown that malaria in early pregnancy is associated with reduced fetal growth velocity regardless of the time elapsed between detection and velocity assessment, suggesting an effect that persists even after treatment [22]. Malaria in early pregnancy inhibits trophoblast invasion, which occurs from very early pregnancy until 18 to 20 weeks’ gestation and is essential for appropriate placental function and fetal growth [19, 37, 38]. Both falciparum and vivax malaria detected before 4 weeks’ gestation increased the odds of SGA considerably, though the confidence intervals were wide and crossed the null. Additionally, some evidence of an association between vivax malaria detected between 12 and 20 weeks’ and SGA was observed after stratifying by symptoms. In Brazil, symptomatic vivax malaria before 20 weeks’ gestation was associated with reduced gestational age and birthweight [20]. This analysis adds to a growing body of evidence that — if the fetus survives [31] — falciparum and vivax malaria in early pregnancy, even when treated, are associated with adverse birth outcomes [19, 20, 31, 39]. This is a problem, because current interventions for malaria in pregnancy — intermittent preventative treatment with sulphadoxine-pyrimethamine (IPTp-SP) and distribution of bed nets at antenatal clinics — do not start until the second trimester.

Several previous studies have found that the effect of falciparum malaria in reducing birthweight increases with the number of episodes [11, 14–16], and one study in Africa found that the effect of falciparum malaria on intrauterine growth restriction was also cumulative [23]. In Malawi, there was no association between the number of falciparum malaria episodes and SGA; however, this was in the context of women receiving malaria screening and three-dose IPTp-SP [13]. We found a cumulative effect of the number of malaria episodes on the risk of SGA, for both falciparum and vivax malaria. Surprisingly, this cumulative effect was greater for vivax malaria than falciparum malaria. This is concerning, because the propensity for vivax malaria to relapse is high, occurring in more than half the cases in this region, and 8-aminoquinolines cannot be used in pregnancy (Fig. 2).

Some studies [18, 19], but not all [12, 13, 17], also found an association between falciparum malaria detected and treated during pregnancy and SGA. Sequestration of *P. falciparum*-infected red blood cells (i.e. placental malaria) is associated with fetal growth restriction [40–42]. In this population, placental changes are rarely found in women who have malaria detected and treated early in pregnancy, suggesting placental recovery [43]. There is some evidence that *P. vivax* can also sequester in the placenta but without associated placental inflammation, suggesting that other factors such as maternal anaemia are responsible for vivax-associated SGA [44]. Other studies have found that malaria detected at delivery, but not malaria detected and treated during pregnancy, is associated with preterm birth [12, 17]. However, we found that malaria detected and treated in later pregnancy (but before delivery) was associated with preterm birth; this discrepancy may be explained by differences in transmission and immunity, antenatal clinic screening methods, and statistical approaches. We found that both symptomatic and asymptomatic malaria were associated with very preterm birth and late preterm birth when detected and treated during the gestation window when women are at risk of these outcomes (28–32 and 32–37 weeks, respectively), and one month prior. These results suggest that the risk of malaria-associated preterm birth persists even after adequate antimalarial treatment. These women may benefit from close monitoring, including preterm birth counselling, cervical length monitoring if ultrasound is available, or potentially Bishop’s score (a measure of cervical preparedness for labour) if ultrasound is not possible [45–47].

The inverse relationship in our data between increasing or stable parasitaemia and reduced proportions of episodes that were symptomatic after 16 weeks’ gestation indicates a specific pregnancy-associated increase in the pyrogenic density. High density parasitaemia and a high proportion of episodes that are symptomatic in very early pregnancy (<8 weeks) are due to the disproportionate number of symptomatic women presenting to outpatient clinics before other women would ordinarily present to antenatal care and begin screening. Pregnancy is associated with reduced immunity to malaria, but the mechanism underlying the change in pyrogenic density and the implications for control of the infection are unclear. Another possibility is that differentiation of fever due to normal signs and symptoms of early pregnancy from fever due to malaria is difficult.

Asymptomatic malaria in pregnancy is unlikely to be treated in the absence of screening or presumptive treatment. It is therefore concerning that asymptomatic malaria in pregnancy detected from 12 weeks’ gestation was associated with preterm birth and/or SGA. In this analysis, we were not able to assess the effect of the interval between screens on the effect of malaria on birth outcomes. Nearly one third of women who had malaria in pregnancy screened positive at their first antenatal clinic visit. Women presenting very early may have had sub-patent infections, as parasitaemia was higher for malaria detected at later gestations. More sensitive diagnostic tools could increase the proportion of infections detected and treated at the first antenatal consultation, thereby preventing the persistence of asymptomatic parasitaemia throughout pregnancy and its adverse consequences. However, more research is needed on the effectiveness of malaria screening in pregnancy, especially in areas of low transmission outside of Africa [48]. Furthermore, a considerable proportion of women had both falciparum and vivax malaria during pregnancy, and 53 million women are at risk of both falciparum and vivax malaria in pregnancy each year. We did not include these women in this analysis because of potentially complex interactions between species; the effects of having both falciparum and vivax malaria in pregnancy should be investigated.

## Conclusions

These results have important implications for interventions for malaria in pregnancy globally. The strongest associations between malaria in pregnancy and SGA and preterm birth were for malaria detected in the third trimester, despite treatment. Therefore, third-trimester malaria must be prevented. It is encouraging that malaria treated before 12 weeks’ gestation was generally not associated with preterm birth or SGA (though our confidence intervals for malaria detected before 12 weeks’ gestation were very wide). However, in a previous analysis of SMRU antenatal data, malaria detected and treated in the first trimester was strongly associated with miscarriage [31], and in this analysis, malaria detected and treated from 12 weeks’ gestation was associated with preterm birth and/or SGA, regardless of the presence of symptoms. An ideal chemoprophylaxis would be active against *P. falciparum* and *P. vivax* and be safe to use, and should be commenced, in the first trimester or prior to conception. Early detection and treatment of both asymptomatic and symptomatic malaria needs to start in the first trimester (unlike IPTp-SP in pregnancy), but even with intensive screening and in the absence of symptoms, malaria in pregnancy is still associated with adverse outcomes [49, 50]. Control and elimination efforts in the general population such as early detection by trained health workers and effective treatment, which has been the major single influence on the burden of malaria in pregnancy in this population [51, 52], should be strengthened to improve maternal and child health in malaria endemic areas.

## Additional files

Additional file 1: Summary of studies assessing the influence of the gestational age at malaria detection and the number of malaria episodes on fetal growth and gestational age-related birth outcomes. (DOCX 129 kb)

Additional file 2: Details of antimalarial treatment studies including pregnant women attending Shoklo Malaria Research Unit antenatal clinics between 1986 and 2015. (DOCX 27 kb)

Additional file 3: Directed acyclic graphs to support statistical methods. (DOCX 2048 kb)

Additional file 4: Table version of Fig. 4: the association between the number of malaria episodes in pregnancy and small-for-gestational-age (SGA). (DOCX 118 kb)

Additional file 5: The association between the gestational age at falciparum or vivax malaria detection and treatment and small-for-gestational-age (SGA), with differentiation between symptomatic and asymptomatic malaria. (DOCX 243 kb)

Additional file 6: The association between the gestational age at falciparum or vivax malaria detection and treatment and preterm birth, with differentiation between symptomatic and asymptomatic malaria. (DOCX 230 kb)

Additional file 7: Sub-group analyses to explore the influence of changes over time. (DOCX 322 kb)

## Abbreviations

ANC: Antenatal clinic
CI: Confidence interval
EGA: Estimated gestational age
IPTp-SP: Intermittent preventative treatment with sulphadoxine-pyrimethamine
OR: Odds ratio
SGA: Small-for-gestational-age
SMRU: Shoklo Malaria Research Unit
