# Supplementary material for: Influence of the number and timing of malaria episodes during pregnancy on prematurity and small-for-gestational-age in an area of low transmission
Source: BMC Med. 2017 Jun 21;15:117. doi: 10.1186/s12916-017-0877-6 (PMC5479010; doi:10.1186/s12916-017-0877-6)
Supplement: Supplementary file 1 — Summary of studies assessing the influence of the gestational age at malaria detection and the number of malaria episodes on fetal growth and gestational age-related birth outcomes. (DOCX 129 kb) [file 12916_2017_877_MOESM1_ESM.docx]

**Additional file 1: Summary of studies assessing the influence of the gestational age at malaria detection and the number of malaria episodes on fetal growth and gestational age related birth outcomes**

We searched Scopus for articles published up to March 31 2017, in any language, that addressed the influence of the timing of malaria in pregnancy on fetal growth-related adverse birth outcomes using the query: (TITLE-ABS-KEY ( malaria OR plasmodium ) AND TITLE-ABS-KEY ( pregnan* ) AND TITLE-ABS-KEY ( "number and" OR "number of * infection*" OR "frequency and" OR "frequency of * infection*" OR "multiple * infection*" OR "time of * infection*" OR timing OR "trimester of * infection*" OR "period of * infection*" ) AND TITLE-ABS-KEY ( preterm OR birthweight OR "birth weight" OR *growth* OR outcome* ) ) AND NOT TITLE-ABS-KEY ( "number needed to treat" ). The results of relevant papers are summarised in Table 1 and Table 2.

Table 1. Studies assessing the influence of the gestation time of malaria on fetal growth and gestational age related birth outcomes

| **Study** | **Country** | **Outcome** | **Measure of association** | **Associations between malaria and outcome, by gestation period**  **Estimate (95% CI)** |
| --- | --- | --- | --- | --- |
| Briand *et al.* 2016 [1] | Benin | Fetal growth velocity Z-score | Adjusted mean difference | ‘Early’ (>1 month prior to Z-score assessment): -0.04 (-0.04, -0.03)  ‘Recent’ (within 1 month prior to Z-score assessment): -0.03 (-0.03, -0.02)  ‘Concomitant’ (at Z-score assessment): -0.05 (-0.06 to -0.04) |
| Kalilani *et al.* 2010 [2] | Malawi | LBW | Adjusted prevalence ratio | Second trimester: 1.71 (1.06, 2.74)  Third trimester: 1.55 (0.88, 2.75) |
| Verhoeff *et al.* 2001 [3] | Malawi | SGA | Odds ratio | At booking: 1.2 (1.0, 1.6)  At delivery: 1.4 (1.1, 1.8) |
|  |  | Preterm | Odds ratio | At booking: 1.0 (0.7, 1.4)  At delivery: 1.3 (1.0, 1.6) |
| Kalilani-Phiri *et al.* 2013 [4] | Malawi | SGA | Odds ratio | During pregnancy: “not associated”  At delivery: 2.2 (1.1, 2.4) |
| Cottrell *et al.* 2007 [5] | Burkina Faso | Birthweight | Adjusted mean difference | <=4 months: -68 (-145, 10)  4-6 months: 18 (-68, 101)  >6 months: -105 (-190, -22) |
| Huynh *et al.* 2011 [6] | Benin | Birthweight | Adjusted mean difference | 0-4 months: -98.5 (-188.5, -8.5)  5-6 months: 35.4 (-41.7, 112.5)  >6 months: -21.9 (-82.2, 38.5) |
| Valea *et al.* 2012 [7] | Burkina Faso | LBW | Adjusted incidence rate ratio | 1^st^ trimester: 2.07 (1.30, 3.27)  2^nd^ trimester: 1.09 (0.65, 1.82)  3^rd^ trimester: 0.78 (0.46, 1.32) |
| Sullivan *et al.* 1999 [8] | Malawi | SGA | Adjusted odds ratio | Antenatal: 1.87 (0.78, 4.78)  At delivery: 1.92 (0.76, 4.81) |
|  |  | Preterm | Adjusted odds ratio | Antenatal: 0.71 (0.35, 1.42)  At delivery: 2.91 (1.36, 6.26) |
| McClure *et al.* 2014 [9] | Kenya | Fetal weight | Adjusted mean difference | 18-20: –36 (–86, 15)  21-23: 16 (–43, 77)  24-26: –22 (–84, 38)  27-29: –31 (–94, 33)  30-32: –123 (–235, –12)  33-35: –13 (–171, 144) |
| Griffin *et al.* 2012 [10] | DRC | Subsequent IUGR episode | Adjusted risk ratio | <=20 weeks’ gestation: 1.8 (1.1, 2.9) |
|  |  | Repeat IUGR episodes | Adjusted risk ratio | <=20 weeks’ gestation: 2.2 (1.1, 4.2) |
| Filho *et al.* 2014 [11] | Brazil | Gestational age | None | <20 weeks’ gestation: malaria 37 (25–41); no malaria 39 (36–42). |
|  |  | Birthweight | None | <20 weeks’ gestation: malaria 2,948.2 ± 789.9; no malaria 3,382.5 ± 289.5 |
| McGready *et al.* 2002 [12] | Thailand | Gestational age | Descriptive (mean [SD]) | First-trimester falciparum: 39.7 [1]  First-trimester vivax: 39.9 [1]  Mo malaria: 39.7 [1.29] |
|  |  | Birthweight | Descriptive (mean [SD]) | First-trimester falciparum: 2882 [572]  First-trimester vivax: 2949 [355]  No malaria: 2931 [473] |
| Nosten *et al.* 1999 [13] | Thailand | Birthweight | Correlation coefficient | Correlation between gestational age of infection and birthweight: 0.026 |

LBW: low birthweight; IUGR: intrauterine growth restriction; SGA: small-for-gestational-age. All studies were of falciparum malaria in Africa, except for Filho *et al.* 2014 (vivax malaria in Brazil), McGready *et al.* 2002 (falciparum and vivax malaria in Thailand), and Nosten *et al.* 1999 (vivax malaria in Thailand).

Table 2. Studies assessing the influence of the number of malaria episodes on fetal growth and gestational age related birth outcomes

| **Study** | **Country** | **Outcome** | **Measure of association** | **Associations between number of episodes and outcome**  **Estimate (95% CI)** |
| --- | --- | --- | --- | --- |
| Briand *et al.* 2016 [1] | Benin | Birthweight for gestational age Z-score | Adjusted mean difference | 1 episode: −0.08 (−.26 to .09)  2+ episodes: −0.19 (−.39 to .02) |
| Landis *et al.* 2009 [14] | DRC | IUGR | Adjusted risk ratio | 1 episode: 0.8 (0.4, 1.5)  2 episodes: 1.6 (0.8, 3.3)  3+ episodes: 3.3 (1.3, 8.2) |
| Kalilani *et al.* 2010 [2] | Malawi | LBW | Adjusted prevalence ratio | 1 episode: 1.62 (1.07, 2.46)  2+ episodes: 2.41 (0.87, 2.54) |
| Kalilani-Phiri *et al.* 2013 [4] | Malawi | SGA | Odds ratio | “Number of malaria episodes were not associated with adverse outcomes.” |
| Cottrell *et al.* 2007 [5] | Burkina Faso | LBW | Adjusted odds ratio | 1-2 episodes: 1.4 (0.9, 2.2)  >2 episodes 2.6 (1.2, 5.7) |
| Huynh *et al.* 2011 [6] | Benin | Birthweight | Adjusted mean difference | 1-2 episodes: −50.1 (−111.6, 11.4)  3+ episodes: −116.4 (−245, 12.6) |
| Valea *et al.* 2012 [7] | Burkina Faso | LBW | Adjusted incidence rate ratio | 1 episode: 1.07 (0.73, 1.58)  2+ episodes: 1.08 (0.62, 1.87) |
| De Beaudrap *et al.* 2013 [15] | Uganda | Birthweight | Adjusted mean difference | 1 episode: non-significant reduction  2+ episodes: greater and significant reduction |
| Nosten *et al.* 1991 [16] | Thailand | Birthweight | Descriptive (mean [SD]) | 5+ episodes: 2.56 kg [0.49]  1 episode: 2.85 kg [0.55] |

LBW: low birthweight; IUGR: intrauterine growth restriction; SGA: small-for-gestational-age. All studies were of falciparum malaria in Africa, except for Nosten *et al.* 1991 (malaria [80% falciparum; 17% vivax; 3% mixed] in Thailand).

**References**

1. Briand V, Saal J, Ghafari C, Huynh B, Fievet N, Schmiegelow C, et al. Fetal growth restriction is associated with malaria in pregnancy: a prospective longitudinal study in Benin. J Infect Dis. 2016;214:417–25.

2. Kalilani L, Mofolo I, Chaponda M, Rogerson SJ, Meshnick SR. The effect of timing and frequency of Plasmodium falciparum infection during pregnancy on the risk of low birth weight and maternal anemia. Trans R Soc Trop Med Hyg. 2010;104:416–22.

3. Verhoeff FH, Brabin BJ, van Buuren S, Chimsuku L, Kazembe P, Wit JM, et al. An analysis of intra-uterine growth retardation in rural Malawi. Eur J Clin Nutr. 2001;55:682–9.

4. Kalilani-Phiri L, Thesing PC, Nyirenda OM, Mawindo P, Madanitsa M, Membe G, et al. Timing of malaria infection during pregnancy has characteristic maternal, infant and placental outcomes. PLoS One. 2013;8:e74643.

5. Cottrell G, Mary J-Y, Barro D, Cot M. The importance of the period of malarial infection during pregnancy on birth weight in tropical Africa. Am J Trop Med Hyg. 2007;76:849–54.

6. Huynh B-T, Fievet N, Gbaguidi G, Dechavanne S, Borgella S, Guézo-Mévo B, et al. Influence of the timing of malaria infection during pregnancy on birth weight and on maternal anemia in Benin. Am J Trop Med Hyg. 2011;85:214–20.

7. Valea I, Tinto H, Drabo MK, Huybregts L, Sorgho H, Ouedraogo J-B, et al. An analysis of timing and frequency of malaria infection during pregnancy in relation to the risk of low birth weight, anaemia and perinatal mortality in Burkina Faso. Malar J. 2012;11:71.

8. Sullivan AD, Nyirenda T, Cullinan T, Taylor T, Harlow SD, James SA, et al. Malaria infection during pregnancy: intrauterine growth retardation and preterm delivery in Malawi. J Infect Dis. 1999;179:1580–3.

9. McClure EM, Meshnick SR, Lazebnik N, Mungai P, King CL, Hudgens M, et al. A cohort study of Plasmodium falciparum malaria in pregnancy and associations with uteroplacental blood flow and fetal anthropometrics in Kenya. Int J Gynecol Obstet. 2014;126:78–82.

10. Griffin JB, Lokomba V, Landis SH, Thorp JM, Herring AH, Tshefu AK, et al. Plasmodium falciparum parasitaemia in the first half of pregnancy, uterine and umbilical artery blood flow, and foetal growth: a longitudinal Doppler ultrasound study. Malar J. 2012;11:319.

11. Machado Filho AC, Da Costa EP, Da Costa EP, Reis IS, Fernandes EAC, Paim B V., et al. Effects of vivax malaria acquired before 20 weeks of pregnancy on subsequent changes in fetal growth. Am J Trop Med Hyg. 2014;90:371–6.

12. McGready R, Thwai K, Cho T, Samuel, Looareesuwan S, White N, et al. The effects of quinine and chloroquine antimalarial treatments in the first trimester of pregnancy. Trans R Soc Trop Med Hyg. 2002;96:180–4.

13. Nosten F, McGready R, Simpson JA, Thwai KL, Balkan S, Cho T, et al. Effects of Plasmodium vivax malaria in pregnancy. Lancet. 1999;354:546–9.

14. Landis SH, Lokomba V, Ananth C V, Atibu J, Ryder RW, Hartmann KE, et al. Impact of maternal malaria and under-nutrition on intrauterine growth restriction: a prospective ultrasound study in Democratic Republic of Congo. Epidemiol Infect. 2009;137:294–304.

15. De Beaudrap P, Turyakira E, White LJ, Nabasumba C, Tumwebaze B, Muehlenbachs A, et al. Impact of malaria during pregnancy on pregnancy outcomes in a Ugandan prospective cohort with intensive malaria screening and prompt treatment. Malar J. 2013;12:139.

16. Nosten F, ter Kuile F, Maelankirri L, Decludt B, White NJ. Malaria during pregnancy in an area of unstable endemicity. Trans R Soc Trop Med Hyg. 1991;85:424–9.
