# Supplementary material for: Influence of the number and timing of malaria episodes during pregnancy on prematurity and small-for-gestational-age in an area of low transmission
Source: BMC Med. 2017 Jun 21;15:117. doi: 10.1186/s12916-017-0877-6 (PMC5479010; doi:10.1186/s12916-017-0877-6)
Supplement: Supplementary file 2 — Details of antimalarial treatment studies including pregnant women attending Shoklo Malaria Research Unit antenatal clinics between 1986 and 2015. (DOCX 27 kb) [file 12916_2017_877_MOESM2_ESM.docx]

**Additional file 2: Details of antimalarial treatment studies including pregnant women attending Shoklo Malaria Research Unit antenatal clinics between 1986 and 2015**

**Table 1. Details of antimalarial treatment studies including pregnant women attending Shoklo Malaria Research Unit antenatal clinics**

| **Study** | **Antimalarial** | **Number of pregnant women** | **Reference** |
| --- | --- | --- | --- |
| **Randomized controlled trials** | | | |
| Randomized comparison of mefloquine-artesunate versus quinine in the treatment of multidrug-resistant falciparum malaria in pregnancy | Mefloquine-artesunate | 66 | [1] |
|  | Quinine | 42 |  |
| Randomized comparison of quinine-clindamycin versus artesunate in the treatment of falciparum malaria in pregnancy | Artesunate | 64 | [2] |
|  | Quinine plus clindamycin | 65 |  |
| A randomized comparison of artesunate-atovaquone-proguanil versus quinine in treatment for uncomplicated falciparum malaria during pregnancy | Atovaquone-Proguanil | 39 | [3] |
|  | Quinine | 42 |  |
| A randomised controlled trial of artemether-lumefantrine versus artesunate for uncomplicated plasmodium falciparum treatment in pregnancy | Artemether-lumefantrine | 125 | [4] |
|  | Artesunate monotherapy | 128 |  |
| Randomized controlled trial of Randomised Trial of 3 Artemisinin Combination Therapies for Malaria in Pregnancy (DMA) | Mefloquine-artesunate | 123 | [Registered trial](https://clinicaltrials.gov/ct2/show/NCT01054248), not yet published |
|  | Artemether-lumefantrine | 125 |  |
|  | Dihyroartemisinin-piperaquine | 122 |  |
| **Pharmacokinetic studies** | | | |
| Artesunate/dihydroartemisinin pharmacokinetics in acute falciparum malaria in pregnancy: absorption, bioavailability, disposition and disease effects | Artesunate | 20 | [5] |
| The pharmacokinetics of atovaquone and proguanil in pregnant women with acute falciparum malaria | Atovaquone-proguanil | 24 | [6] |
| Pharmacokinetics of amodiaquine and desethylamodiaquine in pregnant and postpartum women with Plasmodium vivax malaria | Amodiaquine | 24 | [7] |
| Pharmacokinetics of dihydroartemisinin and piperaquine in pregnant and nonpregnant women with uncomplicated falciparum malaria | Dihydroartemisinin piperaquine | 24 | [8] |
| **Rescue treatment studies** | | | |
| Artemisinin derivatives in the treatment of falciparum malaria in pregnancy | Artesunate | 58 | [9] |
|  | Mefloquine-artesunate | 29 |  |
|  | Artemether | 3 |  |
| Artesunate-atovaquone-proguanil rescue treatment of multidrug-resistant Plasmodium falciparum malaria in pregnancy: a preliminary report | Artesunate-atovaquone-proguanil | 27 | [10] |
| Dihydroartemisinin-piperaquine rescue treatment of multidrug-resistant Plasmodium falciparum malaria in pregnancy: a preliminary report. | Dihydroartemisinin piperaquine | 50 | [11] |
| **Total number of pregnant women included in treatment studies:** | | **1200** |  |

**References**

1. McGready R, Brockman A, Cho T, Cho D, Van Vugt M, Luxemburger C, et al. Randomized comparison of mefloquine-artesunate versus quinine in the treatment of multidrug-resistant falciparum malaria in pregnancy. Trans R Soc Trop Med Hyg. 2000;94:689–93.

2. Mcgready R et al. Randomized comparison of quinine-clindamycin treatment of falciparum malaria in pregnancy versus attesunate in the. Trans R Soc Trop Med Hyg. 2001;95:651–6.

3. McGready R, Ashley EA, Moo E, Cho T, Barends M, Hutagalung R, et al. A randomized comparison of artesunate-atovaquone-proguanil versus quinine in treatment for uncomplicated falciparum malaria during pregnancy. J Infect Dis. 2005;192:846–53. doi:10.1086/432551.

4. McGready R, Tan SO, Ashley E a, Pimanpanarak M, Viladpai-Nguen J, Phaiphun L, et al. A randomised controlled trial of artemether-lumefantrine versus artesunate for uncomplicated plasmodium falciparum treatment in pregnancy. PLoS Med. 2008;5:e253. doi:10.1371/journal.pmed.0050253.

5. McGready R, Phyo AP, Rijken MJ, Tarning J, Lindegardh N, Hanpithakpon W, et al. Artesunate/dihydroartemisinin pharmacokinetics in acute falciparum malaria in pregnancy: absorption, bioavailability, disposition and disease effects. Br J Clin Pharmacol. 2011;73:467–77. doi:10.1111/j.1365-2125.2011.04103.x.

6. McGready R, Stepniewska K, Edstein MD, Cho T, Gilveray G, Looareesuwan S, et al. The pharmacokinetics of atovaquone and proguanil in pregnant women with acute falciparum malaria. Eur J Clin Pharmacol. 2003;59:545–52.

7. Rijken MJ, McGready R, Jullien V, Tarning J, Lindegardh N, Phyo AP, et al. Pharmacokinetics of amodiaquine and desethylamodiaquine in pregnant and postpartum women with Plasmodium vivax malaria. Antimicrob Agents Chemother. 2011;55:4338–42.

8. Rijken MJ, McGready R, Phyo AP, Lindegardh N, Tarning J, Laochan N, et al. Pharmacokinetics of Dihydroartemisinin and Piperaquine in Pregnant and Nonpregnant Women with Uncomplicated Falciparum Malaria. Antimicrob Agents Chemother. 2011;55:5500–6. doi:10.1128/AAC.05067-11.

9. McGready R, Cho T, Cho JJ, Simpson JA, Luxemburger C, Dubowitz L, et al. Artemisinin derivatives in the treatment of falciparum malaria in pregnancy. Trans R Soc Trop Med Hyg. 1998;92:430–3.

10. McGready R, Keo NK, Villegas L, White NJ, Looareesuwan S, Nosten F. Aresunate-atovaquone-proguanil rescue treatment of multidrug-resistant Plasmodium falciparum malaria in pregnancy: a preliminary report. Trans R Soc Trop Med Hyg. 2003;97:592–4.

11. Rijken MJ, McGready R, Boel ME, Barends M, Proux S, Pimanpanarak M, et al. Dihydroartemisinin-piperaquine rescue treatment of multidrug-resistant Plasmodium falciparum malaria in pregnancy: a preliminary report. Am J Trop Med Hyg. 2008;78:543–5. doi:78/4/543 [pii].
