# Supplementary material for: Influence of the number and timing of malaria episodes during pregnancy on prematurity and small-for-gestational-age in an area of low transmission
Source: BMC Med. 2017 Jun 21;15:117. doi: 10.1186/s12916-017-0877-6 (PMC5479010; doi:10.1186/s12916-017-0877-6)
Supplement: Supplementary file 3 — Directed acyclic graphs to support statistical methods. (DOCX 2048 kb) [file 12916_2017_877_MOESM3_ESM.docx]

**Additional file 3: Directed acyclic graphs to support statistical methods**

Figure 1. Directed acyclic graph for the association between the gestation time of malaria detection and treatment and preterm birth. Black arrows indicate associations of interest. Baseline confounders: gravidity (primi- or multi-gravid), clinic site (migrant or refugee), and yearly malaria incidence (quartiles). Very preterm birth: delivery between 28 and 32 weeks’ gestation. Late preterm birth: delivery between 32 and 37 weeks’ gestation. The 9 time intervals for malaria detection and treatment were: <4, ≥4 and <8, ≥8 and <12, ≥12 and <16, ≥16 and <20, ≥20 and <24, ≥24 and <28, ≥28 and <32, ≥32 and <37 weeks’ gestation. Women who gave birth between 28 and 32 weeks’ gestation (time interval 8) did not have an observation for malaria between 32 and 37 weeks’ gestation (time interval 9). Analyses were adjusted for malaria history within the current pregnancy, gravidity, clinic site, and yearly malaria incidence; malaria history refers to malaria at all previous times *t*-x, not just time *t*-1 (arrows not included in diagram for simplicity). Models also included malaria at all subsequent times *t*+x, thereby removing mediation of associations between malaria at time *t* and adverse outcomes through malaria at times *t*+x.

Figure 2. Directed acyclic graph for the association between the gestation time of malaria detection and treatment and small-for-gestational age (SGA). Black arrows indicate associations of interest. Baseline confounders: gravidity (primi- or multi-gravid), clinic site (migrant or refugee), and yearly malaria incidence (quartiles). The 10 time intervals for malaria detection and treatment were: <4, ≥4 and <8, ≥8 and <12, ≥12 and <16, ≥16 and <20, ≥20 and <24, ≥24 and <28, ≥28 and <32, ≥32 and <37, and ≥37 weeks’ gestation. Analyses were adjusted for malaria history within the current pregnancy, gravidity, clinic site, and yearly malaria incidence; malaria history refers to malaria at all previous times *t*-x, not just time *t*-1 (arrows not included in diagram for simplicity). Models also included malaria at all subsequent times *t*+x, thereby removing mediation of associations between malaria at time *t* and adverse outcomes through malaria at times *t*+x.

**Figure 3. Directed acyclic graph for the association between the total number of malaria episodes in pregnancy and small-for-gestational-age (SGA).** The black arrow indicates the association of interest. Baseline confounders: gravidity (primi- or multi-gravid), clinic site (migrant or refugee), and yearly malaria incidence. The 8 time intervals for malaria detection and treatment were: <4, ≥4 and <8, ≥8 and <12, ≥12 and <16, ≥16 and <20, ≥20 and <24, ≥24 and <28, ≥28 weeks’ gestation. Analyses were adjusted for malaria history within the current pregnancy, gravidity, clinic site, and yearly malaria incidence; malaria history refers to malaria at all previous times *t*-x. Adjusting for malaria history removes the effect of the gestation time at which malaria was detected and treated, leaving only the effect of the total number of malaria episodes in pregnancy.


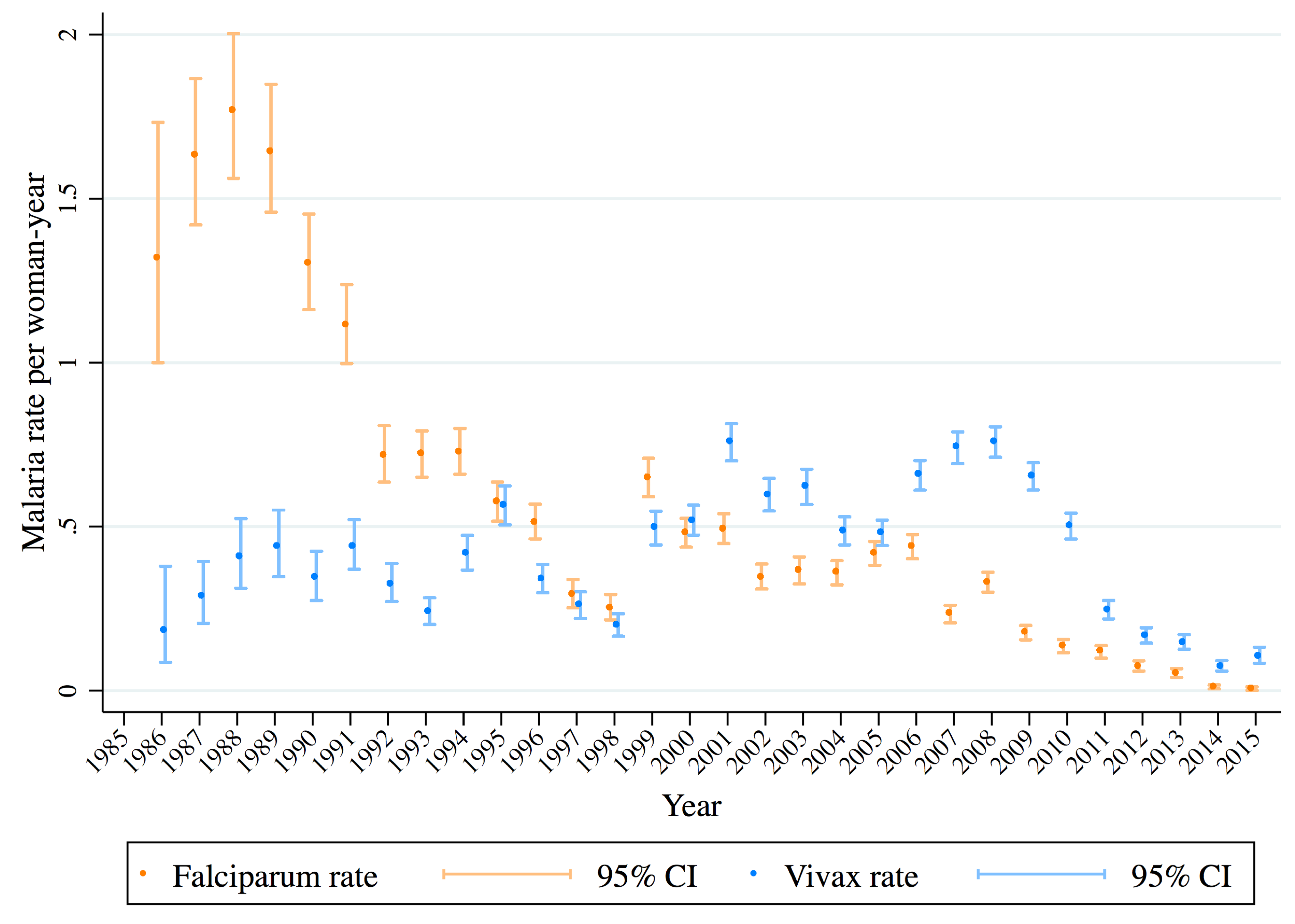


Figure 4. Incidence rate of falciparum and vivax malaria in pregnancy per woman-year between 1986 and 2015 in women attending SMRU antenatal clinics. Time at risk of malaria in pregnancy was considered as the time between a woman’s first antenatal consultation and delivery or time last seen (which ever came first). Women may contribute more than one malaria episode.
