# Supplementary material for: Influence of the number and timing of malaria episodes during pregnancy on prematurity and small-for-gestational-age in an area of low transmission
Source: BMC Med. 2017 Jun 21;15:117. doi: 10.1186/s12916-017-0877-6 (PMC5479010; doi:10.1186/s12916-017-0877-6)
Supplement: Supplementary file 4 — Table version of Fig. 4: the association between the number of malaria episodes in pregnancy and small-for-gestational-age (SGA). (DOCX 118 kb) [file 12916_2017_877_MOESM4_ESM.docx]

Additional file 4: The association between the number of malaria episodes in pregnancy and small-for-gestational-age

Table. Table version of Figure 4 - The association between the number of malaria episodes in pregnancy and small-for-gestational-age (SGA).

|  | **Unadjusted OR [95% CI]; p-value** | **Adjusted OR [95% CI]; p-value** | **SGA (%)** |
| --- | --- | --- | --- |
| **Falciparum** |  |  |  |
| No malaria | Reference Group | Reference Group | 7965 (20) |
| 1 episode | 1.30 [1.21, 1.41]; <0.001 | 1.17 [1.07, 1.28]; 0.001 | 931 (25) |
| 2 episodes | 1.53 [1.37, 1.72]; <0.001 | 1.33 [1.16, 1.52]; <0.001 | 418 (28) |
| 3 episodes | 1.71 [1.45, 2.02]; <0.001 | 1.48 [1.23, 1.79]; <0.001 | 209 (30) |
| 4 episodes | 1.60 [1.28, 2.01]; <0.001 | 1.45 [1.13, 1.86]; 0.003 | 108 (29) |
| 5 episodes | 1.98 [1.51, 2.61]; <0.001 | 1.81 [1.33, 2.46]; <0.001 | 77 (33) |
| 6 episodes | 2.32 [1.71, 3.16]; <0.001 | 2.18 [1.53, 3.11]; <0.001 | 64 (37) |
| Per episode^*^ |  | 1.13 [1.09, 1.17]; <0.001 | NA |
| Per symptomatic episode^*#^ |  | 1.17 [1.11, 1.24]; <0.001 | NA |
| Per asymptomatic episode^*^^ |  | 1.10 [1.05, 1.15]; <0.001 | NA |
| **Vivax** |  |  |  |
| No malaria | Reference Group | Reference Group | 7965 (20) |
| 1 episode | 1.32 [1.22, 1.43]; <0.001 | 1.40 [1.28, 1.53]; <0.001 | 897 (25) |
| 2 episodes | 1.50 [1.33, 1.68]; <0.001 | 1.70 [1.48, 1.96]; <0.001 | 408 (27) |
| 3 episodes | 1.61 [1.38, 1.89]; <0.001 | 2.05 [1.68, 2.50]; <0.001 | 218 (29) |
| 4 episodes | 1.63 [1.32, 2.00]; <0.001 | 2.37 [1.82, 3.08]; <0.001 | 130 (29) |
| 5 episodes | 1.81 [1.40, 2.35]; <0.001 | 2.81 [2.04, 3.86]; <0.001 | 84 (31) |
| 6 episodes | 2.32 [1.73, 3.12]; <0.001 | 4.11 [2.79, 6.05]; <0.001 | 71 (37) |
| Per episode^*^ |  | 1.27 [1.21, 1.33]; <0.001 | NA |
| Per symptomatic episode^*#^ |  | 1.28 [1.21, 1.35]; <0.001 | NA |
| Per asymptomatic episode^*^^ |  | 1.24 [1.17, 1.31]; <0.001 | NA |

Numbers are odds ratios (OR) [95% confidence interval]; p-value. Models were adjusted for gravidity; clinic site; yearly malaria incidence; and malaria history within the current pregnancy (see Figure S3). **p*-values for evidence against the null hypothesis that the association between the number of malaria episodes and the log odds of SGA is linear: falciparum 0.819; symptomatic falciparum 0.114; asymptomatic falciparum 0.359; vivax 0.118; symptomatic vivax 0.593; asymptomatic vivax 0.155. ^#^Model is also adjusted for the number of asymptomatic malaria episodes. ^^^Model is also adjusted for the number of symptomatic malaria episodes. Confounding mostly due to malaria history (falciparum and vivax models), and yearly malaria incidence (falciparum model only).
