# Supplementary material for: Influence of the number and timing of malaria episodes during pregnancy on prematurity and small-for-gestational-age in an area of low transmission
Source: BMC Med. 2017 Jun 21;15:117. doi: 10.1186/s12916-017-0877-6 (PMC5479010; doi:10.1186/s12916-017-0877-6)
Supplement: Supplementary file 5 — The association between the gestational age at falciparum or vivax malaria detection and treatment and small-for-gestational-age (SGA), with differentiation between symptomatic and asymptomatic malaria. (DOCX 243 kb) [file 12916_2017_877_MOESM5_ESM.docx]

**Additional file 5: The association between the gestational age at falciparum or vivax malaria detection and treatment and small-for-gestational-age (SGA), with differentiation between symptomatic and asymptomatic malaria.**

Figure. The association between the gestational age at falciparum or vivax malaria detection and treatment and small-for-gestational-age (SGA), with differentiation between symptomatic and asymptomatic malaria. Orange circles: falciparum malaria; blue circles: vivax malaria. Models were adjusted for gravidity; clinic site; yearly malaria incidence (quartiles); and malaria history within the current pregnancy (see Figure S2). See Table S3 for a table version of this figure, including univariable associations. The associations between asymptomatic falciparum and vivax malaria detected at 0-4 weeks’ gestation and SGA were not plotted due to too few events in the malaria groups (see Table 5 of supplementary material). The reference group for a/symptomatic malaria at time *t* is no malaria (either asymptomatic or symptomatic) detected at time *t.*

Table. Table version of Figure 5 and Additional file 5 figure (above) - the association between the gestational age at falciparum or vivax malaria detection and treatment and small-for-gestational-age (SGA), with differentiation between symptomatic and asymptomatic malaria.

| **Timing** | **Unadjusted OR [95% CI]; *p*-value** | **Adjusted OR [95% CI]; *p*-value** | **SGA** | **AGA** |
| --- | --- | --- | --- | --- |
| **Falciparum (all levels of severity)** | | | | |
| No malaria^*^ | Reference Group | Reference Group | 7965 (20) | 31796 (80) |
| 0-4 weeks | 1.45 [0.92, 2.28]; 0.113 | 1.43 [0.91, 2.26]; 0.125 | 27 (30) | 63 (70) |
| 4-8 weeks | 0.82 [0.58, 1.17]; 0.281 | 0.84 [0.59, 1.20]; 0.336 | 42 (22) | 151 (78) |
| 8-12 weeks | 1.11 [0.89, 1.39]; 0.347 | 1.06 [0.85, 1.33]; 0.592 | 118 (26) | 332 (74) |
| 12-16 weeks | 1.36 [1.13, 1.63]; 0.001 | 1.26 [1.04, 1.52]; 0.016 | 176 (30) | 410 (70) |
| 16-20 weeks | 1.26 [1.07, 1.49]; 0.007 | 1.20 [1.01, 1.42]; 0.037 | 213 (29) | 525 (71) |
| 20-24 weeks | 1.25 [1.06, 1.47]; 0.009 | 1.15 [0.98, 1.36]; 0.094 | 229 (29) | 550 (71) |
| 24-28 weeks | 1.31 [1.12, 1.53]; 0.001 | 1.23 [1.05, 1.44]; 0.012 | 252 (30) | 575 (70) |
| 28-32 weeks | 1.43 [1.22, 1.66]; <0.001 | 1.30 [1.12, 1.52]; 0.001 | 280 (32) | 596 (68) |
| 32-37 weeks | 1.54 [1.34, 1.78]; <0.001 | 1.45 [1.26, 1.68]; <0.001 | 313 (32) | 651 (68) |
| 37+ weeks | 1.66 [1.34, 2.04]; <0.001 | 1.63 [1.32, 2.02]; <0.001 | 141 (34) | 271 (66) |
| **Asymptomatic falciparum** | | | | |
| No malaria^*^ | Reference Group | Reference Group | 7965 (20) | 31796 (80) |
| 0-4 weeks | Not estimated | Not estimated | 0 (0) | 1 (100) |
| 4-8 weeks | 0.73 [0.30, 1.78]; 0.485 | 0.71 [0.29, 1.74]; 0.454 | 6 (18) | 28 (82) |
| 8-12 weeks | 1.23 [0.82, 1.83]; 0.312 | 1.16 [0.78, 1.73]; 0.465 | 35 (27) | 94 (73) |
| 12-16 weeks | 1.38 [1.03, 1.86]; 0.032 | 1.23 [0.91, 1.66]; 0.173 | 66 (30) | 155 (70) |
| 16-20 weeks | 1.40 [1.07, 1.84]; 0.016 | 1.28 [0.97, 1.68]; 0.081 | 80 (30) | 183 (70) |
| 20-24 weeks | 1.33 [1.04, 1.70]; 0.023 | 1.15 [0.90, 1.47]; 0.270 | 99 (30) | 230 (70) |
| 24-28 weeks | 1.51 [1.21, 1.90]; <0.001 | 1.32 [1.05, 1.66]; 0.017 | 120 (33) | 248 (67) |
| 28-32 weeks | 1.40 [1.12, 1.74]; 0.003 | 1.21 [0.97, 1.51]; 0.096 | 122 (31) | 273 (69) |
| 32-37 weeks | 1.55 [1.24, 1.92]; <0.001 | 1.39 [1.11, 1.73]; 0.003 | 130 (32) | 272 (68) |
| 37+ weeks | 1.68 [1.24, 2.29]; 0.001 | 1.64 [1.20, 2.23]; 0.002 | 63 (34) | 124 (66) |
| **Symptomatic falciparum** | | | | |
| No malaria^*^ | Reference Group | Reference Group | 7965 (20) | 31796 (80) |
| 0-4 weeks | 1.48 [0.94, 2.34]; 0.093 | 1.45 [0.92, 2.29]; 0.114 | 27 (30) | 62 (70) |
| 4-8 weeks | 0.92 [0.63, 1.34]; 0.676 | 0.93 [0.64, 1.35]; 0.687 | 37 (23) | 126 (77) |
| 8-12 weeks | 1.12 [0.87, 1.45]; 0.392 | 1.05 [0.81, 1.36]; 0.712 | 85 (26) | 241 (74) |
| 12-16 weeks | 1.44 [1.15, 1.81]; 0.002 | 1.33 [1.06, 1.68]; 0.014 | 114 (30) | 260 (70) |
| 16-20 weeks | 1.24 [1.01, 1.53]; 0.038 | 1.18 [0.96, 1.45]; 0.124 | 136 (28) | 348 (72) |
| 20-24 weeks | 1.31 [1.06, 1.62]; 0.011 | 1.24 [1.00, 1.53]; 0.048 | 134 (29) | 324 (71) |
| 24-28 weeks | 1.27 [1.03, 1.56]; 0.024 | 1.21 [0.98, 1.49]; 0.071 | 138 (29) | 333 (71) |
| 28-32 weeks | 1.57 [1.29, 1.91]; <0.001 | 1.46 [1.20, 1.78]; <0.001 | 164 (33) | 332 (67) |
| 32-37 weeks | 1.64 [1.37, 1.96]; <0.001 | 1.56 [1.30, 1.87]; <0.001 | 192 (33) | 388 (67) |
| 37+ weeks | 1.72 [1.30, 2.28]; <0.001 | 1.67 [1.26, 2.21]; <0.001 | 78 (35) | 147 (65) |
| **Vivax (all levels of severity)** | | | | |
| No malaria^*^ | Reference Group | Reference Group | 7965 (20) | 31796 (80) |
| 0-4 weeks | 1.60 [0.91, 2.80]; 0.103 | 1.54 [0.88, 2.72]; 0.131 | 19 (34) | 37 (66) |
| 4-8 weeks | 1.01 [0.76, 1.34]; 0.942 | 1.01 [0.76, 1.33]; 0.966 | 72 (25) | 214 (75) |
| 8-12 weeks | 1.15 [0.94, 1.40]; 0.166 | 1.13 [0.92, 1.38]; 0.239 | 156 (28) | 404 (72) |
| 12-16 weeks | 0.95 [0.79, 1.14]; 0.564 | 0.99 [0.82, 1.18]; 0.877 | 188 (25) | 573 (75) |
| 16-20 weeks | 1.03 [0.87, 1.21]; 0.758 | 1.06 [0.90, 1.25]; 0.484 | 230 (26) | 662 (74) |
| 20-24 weeks | 1.10 [0.94, 1.28]; 0.244 | 1.13 [0.96, 1.32]; 0.138 | 268 (26) | 754 (74) |
| 24-28 weeks | 1.20 [1.04, 1.39]; 0.015 | 1.20 [1.04, 1.40]; 0.014 | 317 (28) | 829 (72) |
| 28-32 weeks | 1.10 [0.96, 1.27]; 0.172 | 1.12 [0.97, 1.29]; 0.121 | 315 (26) | 913 (74) |
| 32-37 weeks | 1.27 [1.13, 1.44]; <0.001 | 1.32 [1.17, 1.49]; <0.001 | 460 (28) | 1,212 (72) |
| 37+ weeks | 1.45 [1.24, 1.70]; <0.001 | 1.54 [1.31, 1.81]; <0.001 | 253 (31) | 574 (69) |
| **Asymptomatic vivax** | | | | |
| No malaria^*^ | Reference Group | Reference Group | 7965 (20) | 31796 (80) |
| 0-4 weeks | 1.92 [0.17, 21.13]; 0.596 | 2.05 [0.18, 23.38]; 0.565 | 1 (33) | 2 (67) |
| 4-8 weeks | 1.11 [0.73, 1.70]; 0.623 | 1.14 [0.74, 1.74]; 0.553 | 30 (26) | 87 (74) |
| 8-12 weeks | 1.33 [1.02, 1.73]; 0.033 | 1.31 [1.01, 1.71]; 0.044 | 87 (30) | 207 (70) |
| 12-16 weeks | 0.90 [0.71, 1.14]; 0.388 | 0.95 [0.75, 1.21]; 0.696 | 101 (23) | 338 (77) |
| 16-20 weeks | 0.93 [0.75, 1.15]; 0.493 | 0.96 [0.77, 1.19]; 0.704 | 125 (23) | 411 (77) |
| 20-24 weeks | 1.10 [0.90, 1.33]; 0.352 | 1.12 [0.93, 1.36]; 0.239 | 161 (25) | 479 (75) |
| 24-28 weeks | 1.28 [1.07, 1.52]; 0.006 | 1.28 [1.07, 1.53]; 0.006 | 204 (28) | 531 (72) |
| 28-32 weeks | 1.00 [0.84, 1.20]; 0.969 | 1.03 [0.86, 1.23]; 0.779 | 180 (23) | 598 (77) |
| 32-37 weeks | 1.31 [1.14, 1.52]; <0.001 | 1.35 [1.17, 1.56]; <0.001 | 299 (27) | 789 (73) |
| 37+ weeks | 1.48 [1.23, 1.79]; <0.001 | 1.56 [1.29, 1.89]; <0.001 | 168 (30) | 393 (70) |
| **Symptomatic vivax** | | | | |
| No malaria^*^ | Reference Group | Reference Group | 7965 (20) | 31796 (80) |
| 0-4 weeks | 1.62 [0.91, 2.90]; 0.102 | 1.58 [0.88, 2.83]; 0.123 | 18 (34) | 35 (66) |
| 4-8 weeks | 1.00 [0.70, 1.43]; 0.991 | 1.00 [0.69, 1.43]; 0.984 | 42 (25) | 128 (75) |
| 8-12 weeks | 1.08 [0.81, 1.43]; 0.597 | 1.07 [0.80, 1.42]; 0.654 | 70 (26) | 198 (74) |
| 12-16 weeks | 1.15 [0.89, 1.48]; 0.295 | 1.17 [0.90, 1.51]; 0.241 | 87 (27) | 236 (73) |
| 16-20 weeks | 1.32 [1.04, 1.68]; 0.022 | 1.36 [1.07, 1.73]; 0.012 | 105 (29) | 252 (71) |
| 20-24 weeks | 1.24 [0.98, 1.57]; 0.067 | 1.28 [1.01, 1.61]; 0.042 | 109 (28) | 275 (72) |
| 24-28 weeks | 1.16 [0.92, 1.46]; 0.202 | 1.18 [0.94, 1.49]; 0.161 | 113 (27) | 298 (73) |
| 28-32 weeks | 1.40 [1.13, 1.72]; 0.002 | 1.40 [1.13, 1.73]; 0.002 | 137 (30) | 317 (70) |
| 32-37 weeks | 1.23 [1.02, 1.49]; 0.034 | 1.28 [1.05, 1.55]; 0.013 | 162 (27) | 428 (73) |
| 37+ weeks | 1.52 [1.17, 1.98]; 0.002 | 1.62 [1.24, 2.12]; <0.001 | 86 (32) | 182 (68) |

SGA: small-for-gestational-age. AGA: appropriate for gestational age. Numbers are odds ratios [95% confidence interval]; p-value, or N (%). ‘Not estimated’: Model did not estimate this parameter because there were too few events. Adjusted for gravidity; clinic site; yearly malaria incidence (quartiles); and malaria history within the current pregnancy (see Figure S2). ^*^The reference group for malaria at time *t* is no malaria detected at time *t*; the reference group for a/symptomatic malaria at time *t* is no malaria (either asymptomatic or symptomatic) detected at time *t.*
