# Supplementary material for: Influence of the number and timing of malaria episodes during pregnancy on prematurity and small-for-gestational-age in an area of low transmission
Source: BMC Med. 2017 Jun 21;15:117. doi: 10.1186/s12916-017-0877-6 (PMC5479010; doi:10.1186/s12916-017-0877-6)
Supplement: Supplementary file 6 — The association between the gestational age at falciparum or vivax malaria detection and treatment and preterm birth, with differentiation between symptomatic and asymptomatic malaria. (DOCX 230 kb) [file 12916_2017_877_MOESM6_ESM.docx]

**Additional file 6: The association between the gestational age at falciparum or vivax malaria detection and treatment and preterm birth, with differentiation between symptomatic and asymptomatic malaria.**

Figure. The association between the gestational age at falciparum or vivax malaria detection and treatment and preterm birth, with differentiation between symptomatic and asymptomatic malaria. Orange: falciparum; blue circles: vivax. Models were adjusted for gravidity; clinic site; yearly malaria incidence; and malaria history (within the current pregnancy) (see Figure S1). See Table S6 for a table version of this figure, including univariable associations. The reference group for a/symptomatic malaria at time *t* is no malaria (either asymptomatic or symptomatic) detected at time *t.*

Table. Table version of Figure 6 and Additional file 6 Figure (above) - The association between the gestational age at initial falciparum or vivax malaria detection and treatment and preterm birth.

|  | **Very preterm** | | | | **Late preterm** | | | |
| --- | --- | --- | --- | --- | --- | --- | --- | --- |
| **Timing** | **Unadjusted** | **Adjusted** | **Very preterm** | **Term** | **Unadjusted** | **Adjusted** | **Late Preterm** | **Term** |
| **Falciparum (all)** |  |  |  |  |  |  |  |  |
| No malaria | Reference Group | Reference Group | 421 (1) | 37957 (99) | Reference Group | Reference Group | 3461 (8) | 37957 (92) |
| 0-4 weeks | Not estimated | Not estimated | 0 (0) | 82 (100) | 1.41 [0.77, 2.60]; 0.271 | 1.32 [0.71, 2.44]; 0.375 | 12 (13) | 82 (87) |
| 4-8 weeks | 0.43 [0.06, 3.09]; 0.399 | 0.43 [0.06, 3.11]; 0.402 | 1 (1) | 180 (99) | 1.19 [0.78, 1.84]; 0.420 | 1.22 [0.79, 1.88]; 0.370 | 25 (12) | 180 (88) |
| 8-12 weeks | 0.77 [0.28, 2.11]; 0.613 | 0.73 [0.27, 2.01]; 0.547 | 4 (1) | 420 (99) | 1.12 [0.83, 1.51]; 0.468 | 1.03 [0.76, 1.39]; 0.871 | 52 (11) | 420 (89) |
| 12-16 weeks | 0.72 [0.31, 1.65]; 0.437 | 0.68 [0.30, 1.56]; 0.361 | 6 (1) | 562 (99) | 1.09 [0.84, 1.42]; 0.531 | 0.94 [0.72, 1.23]; 0.661 | 68 (11) | 562 (89) |
| 16-20 weeks | 1.07 [0.57, 2.02]; 0.824 | 1.02 [0.54, 1.92]; 0.944 | 11 (2) | 687 (98) | 1.28 [1.02, 1.62]; 0.032 | 1.18 [0.94, 1.49]; 0.154 | 93 (12) | 687 (88) |
| 20-24 weeks | 1.32 [0.78, 2.24]; 0.294 | 1.23 [0.73, 2.09]; 0.431 | 17 (2) | 772 (98) | 0.84 [0.66, 1.08]; 0.172 | 0.72 [0.56, 0.92]; 0.008 | 79 (9) | 772 (91) |
| 24-28 weeks | 2.72 [1.81, 4.09]; <0.001 | 2.53 [1.69, 3.81]; <0.001 | 30 (4) | 792 (96) | 1.14 [0.92, 1.42]; 0.224 | 1.00 [0.80, 1.24]; 0.971 | 107 (12) | 792 (88) |
| 28-32 weeks | 2.21 [1.44, 3.38]; <0.001 | 2.00 [1.30, 3.06]; 0.001 | 26 (3) | 800 (97) | 1.71 [1.42, 2.07]; <0.001 | 1.44 [1.20, 1.75]; <0.001 | 150 (16) | 800 (84) |
| 32-37 weeks | NA | NA | NA | 897 (100) | 1.70 [1.42, 2.03]; <0.001 | 1.46 [1.22, 1.74]; <0.001 | 164 (15) | 897 (85) |
| **Asymptomatic falciparum** |  |  |  |  |  |  |  |  |
| No malaria | Reference Group | Reference Group | 421 (1) | 37957 (99) | Reference Group | Reference Group | 3461 (8) | 37957 (92) |
| 0-4 weeks | Not estimated | Not estimated | 0 (0) | 1 (100) | Not estimated | Not estimated | 0 (0) | 1 (100) |
| 4-8 weeks | Not estimated | Not estimated | 0 (0) | 31 (100) | 1.64 [0.63, 4.25]; 0.310 | 1.61 [0.62, 4.21]; 0.330 | 5 (14) | 31 (86) |
| 8-12 weeks | 0.61 [0.08, 4.39]; 0.620 | 0.56 [0.08, 4.08]; 0.569 | 1 (1) | 126 (99) | 1.06 [0.60, 1.86]; 0.844 | 0.97 [0.55, 1.71]; 0.923 | 14 (10) | 126 (90) |
| 12-16 weeks | 1.00 [0.31, 3.21]; 0.998 | 0.92 [0.29, 2.93]; 0.887 | 3 (1) | 218 (99) | 1.30 [0.87, 1.93]; 0.198 | 1.07 [0.72, 1.59]; 0.749 | 29 (12) | 218 (88) |
| 16-20 weeks | 1.39 [0.55, 3.48]; 0.485 | 1.28 [0.51, 3.21]; 0.600 | 5 (2) | 259 (98) | 1.02 [0.68, 1.53]; 0.918 | 0.88 [0.59, 1.31]; 0.520 | 28 (10) | 259 (90) |
| 20-24 weeks | 1.33 [0.60, 2.94]; 0.486 | 1.16 [0.53, 2.57]; 0.709 | 7 (2) | 341 (98) | 0.90 [0.63, 1.29]; 0.561 | 0.71 [0.49, 1.01]; 0.058 | 35 (9) | 341 (91) |
| 24-28 weeks | 3.14 [1.78, 5.52]; <0.001 | 2.69 [1.52, 4.75]; 0.001 | 14 (4) | 363 (96) | 1.27 [0.93, 1.71]; 0.129 | 1.00 [0.73, 1.35]; 0.977 | 52 (13) | 363 (87) |
| 28-32 weeks | 1.05 [0.46, 2.42]; 0.907 | 0.93 [0.40, 2.13]; 0.857 | 6 (2) | 369 (98) | 1.93 [1.49, 2.51]; <0.001 | 1.50 [1.16, 1.96]; 0.002 | 75 (17) | 369 (83) |
| 32-37 weeks | NA | NA | NA | 402 (100) | 1.51 [1.15, 1.98]; 0.003 | 1.20 [0.91, 1.58]; 0.190 | 67 (14) | 402 (86) |
| **Symptomatic falciparum** |  |  |  |  |  |  |  |  |
| No malaria | Reference Group | Reference Group | 421 (1) | 37957 (99) | Reference Group | Reference Group | 3461 (8) | 37957 (92) |
| 0-4 weeks | Not estimated | Not estimated | 0 (0) | 81 (100) | 1.43 [0.77, 2.63]; 0.255 | 1.32 [0.71, 2.44]; 0.377 | 12 (13) | 81 (87) |
| 4-8 weeks | 0.47 [0.06, 3.44]; 0.459 | 0.46 [0.06, 3.35]; 0.443 | 1 (1) | 153 (99) | 1.15 [0.71, 1.85]; 0.566 | 1.13 [0.70, 1.83]; 0.605 | 20 (12) | 153 (88) |
| 8-12 weeks | 0.83 [0.26, 2.67]; 0.760 | 0.78 [0.25, 2.51]; 0.682 | 3 (1) | 297 (99) | 1.26 [0.90, 1.77]; 0.184 | 1.13 [0.80, 1.59]; 0.487 | 40 (12) | 297 (88) |
| 12-16 weeks | 0.60 [0.19, 1.91]; 0.386 | 0.55 [0.17, 1.74]; 0.307 | 3 (1) | 353 (99) | 1.02 [0.73, 1.43]; 0.921 | 0.87 [0.62, 1.22]; 0.425 | 40 (10) | 353 (90) |
| 16-20 weeks | 0.92 [0.40, 2.12]; 0.844 | 0.88 [0.38, 2.03]; 0.764 | 6 (1) | 438 (99) | 1.44 [1.10, 1.89]; 0.008 | 1.33 [1.01, 1.75]; 0.041 | 65 (13) | 438 (87) |
| 20-24 weeks | 1.51 [0.78, 2.93]; 0.219 | 1.41 [0.73, 2.73]; 0.308 | 10 (2) | 439 (98) | 0.87 [0.63, 1.20]; 0.410 | 0.76 [0.55, 1.05]; 0.096 | 44 (9) | 439 (91) |
| 24-28 weeks | 2.47 [1.45, 4.23]; 0.001 | 2.34 [1.37, 4.01]; 0.002 | 16 (4) | 441 (96) | 1.12 [0.84, 1.50]; 0.428 | 1.02 [0.77, 1.37]; 0.874 | 56 (11) | 441 (89) |
| 28-32 weeks | 3.41 [2.12, 5.48]; <0.001 | 3.03 [1.88, 4.89]; <0.001 | 20 (4) | 445 (96) | 1.57 [1.21, 2.02]; 0.001 | 1.36 [1.06, 1.76]; 0.018 | 76 (15) | 445 (85) |
| 32-37 weeks | NA | NA | NA | 512 (100) | 1.87 [1.50, 2.34]; <0.001 | 1.67 [1.33, 2.09]; <0.001 | 99 (16) | 512 (84) |
| **Vivax (all)** |  |  |  |  |  |  |  |  |
| No malaria | Reference Group | Reference Group | 421 (1) | 37957 (99) | Reference Group | Reference Group | 3461 (8) | 37957 (92) |
| 0-4 weeks | Not estimated | Not estimated | 0 (0) | 52 (100) | 1.13 [0.48, 2.65]; 0.777 | 1.10 [0.47, 2.58]; 0.830 | 6 (10) | 52 (90) |
| 4-8 weeks | 0.83 [0.25, 2.69]; 0.752 | 0.82 [0.25, 2.65]; 0.738 | 3 (1) | 263 (99) | 1.10 [0.73, 1.65]; 0.648 | 1.10 [0.73, 1.65]; 0.651 | 28 (10) | 263 (90) |
| 8-12 weeks | 0.89 [0.38, 2.09]; 0.797 | 0.86 [0.37, 2.01]; 0.727 | 6 (1) | 510 (99) | 1.19 [0.90, 1.59]; 0.225 | 1.18 [0.88, 1.57]; 0.261 | 60 (11) | 510 (89) |
| 12-16 weeks | 1.17 [0.60, 2.25]; 0.647 | 1.17 [0.60, 2.26]; 0.646 | 11 (2) | 714 (98) | 0.85 [0.64, 1.12]; 0.241 | 0.88 [0.67, 1.17]; 0.389 | 63 (8) | 714 (92) |
| 16-20 weeks | 0.75 [0.37, 1.53]; 0.431 | 0.75 [0.37, 1.51]; 0.418 | 9 (1) | 830 (99) | 0.94 [0.73, 1.20]; 0.622 | 0.97 [0.76, 1.25]; 0.832 | 81 (9) | 830 (91) |
| 20-24 weeks | 0.98 [0.52, 1.82]; 0.941 | 0.94 [0.50, 1.75]; 0.844 | 12 (1) | 956 (99) | 1.04 [0.82, 1.30]; 0.753 | 1.05 [0.84, 1.33]; 0.650 | 97 (9) | 956 (91) |
| 24-28 weeks | 1.86 [1.15, 3.00]; 0.011 | 1.79 [1.11, 2.90]; 0.017 | 21 (2) | 1,051 (98) | 1.22 [0.99, 1.51]; 0.068 | 1.22 [0.98, 1.50]; 0.072 | 117 (10) | 1,051 (90) |
| 28-32 weeks | 1.22 [0.72, 2.08]; 0.457 | 1.18 [0.69, 2.02]; 0.536 | 16 (1) | 1,127 (99) | 1.22 [1.00, 1.48]; 0.048 | 1.23 [1.01, 1.50]; 0.039 | 129 (10) | 1,127 (90) |
| 32-37 weeks | NA | NA | NA | 1,585 (100) | 0.88 [0.73, 1.07]; 0.209 | 0.91 [0.75, 1.10]; 0.311 | 143 (8) | 1,585 (92) |
| **Asymptomatic vivax** |  |  |  |  |  |  |  |  |
| No malaria | Reference Group | Reference Group | 421 (1) | 37957 (99) | Reference Group | Reference Group | 3461 (8) | 37957 (92) |
| 0-4 weeks | Not estimated | Not estimated | 0 (0) | 3 (100) | Not estimated | Not estimated | 0 (0) | 3 (100) |
| 4-8 weeks | 1.26 [0.30, 5.32]; 0.750 | 1.23 [0.29, 5.20]; 0.774 | 2 (2) | 107 (98) | 1.08 [0.58, 2.03]; 0.806 | 1.11 [0.59, 2.09]; 0.753 | 11 (9) | 107 (91) |
| 8-12 weeks | 0.79 [0.25, 2.56]; 0.697 | 0.76 [0.23, 2.44]; 0.640 | 3 (1) | 276 (99) | 0.90 [0.58, 1.38]; 0.624 | 0.89 [0.58, 1.37]; 0.599 | 24 (8) | 276 (92) |
| 12-16 weeks | 1.71 [0.84, 3.50]; 0.140 | 1.73 [0.84, 3.53]; 0.134 | 9 (2) | 411 (98) | 0.82 [0.57, 1.19]; 0.295 | 0.88 [0.61, 1.28]; 0.497 | 33 (7) | 411 (93) |
| 16-20 weeks | 0.86 [0.37, 2.00]; 0.723 | 0.85 [0.37, 1.97]; 0.703 | 6 (1) | 496 (99) | 1.13 [0.84, 1.52]; 0.433 | 1.17 [0.87, 1.58]; 0.303 | 53 (10) | 496 (90) |
| 20-24 weeks | 0.87 [0.40, 1.91]; 0.724 | 0.84 [0.38, 1.84]; 0.656 | 7 (1) | 599 (99) | 1.03 [0.77, 1.37]; 0.849 | 1.06 [0.79, 1.41]; 0.716 | 56 (9) | 599 (91) |
| 24-28 weeks | 1.76 [1.00, 3.11]; 0.051 | 1.69 [0.95, 2.98]; 0.073 | 14 (2) | 668 (98) | 1.32 [1.03, 1.69]; 0.030 | 1.31 [1.02, 1.68]; 0.036 | 78 (10) | 668 (90) |
| 28-32 weeks | 1.13 [0.59, 2.17]; 0.721 | 1.09 [0.57, 2.10]; 0.799 | 10 (1) | 729 (99) | 1.09 [0.85, 1.40]; 0.480 | 1.11 [0.86, 1.43]; 0.422 | 74 (9) | 729 (91) |
| 32-37 weeks | NA | NA | NA | 1,040 (100) | 0.79 [0.62, 1.00]; 0.048 | 0.80 [0.63, 1.01]; 0.065 | 82 (7) | 1,040 (93) |
| **Symptomatic vivax** |  |  |  |  |  |  |  |  |
| No malaria | Reference Group | Reference Group | 421 (1) | 37957 (99) | Reference Group | Reference Group | 3461 (8) | 37957 (92) |
| 0-4 weeks | Not estimated | Not estimated | 0 (0) | 49 (100) | 1.11 [0.47, 2.63]; 0.805 | 1.09 [0.46, 2.58]; 0.841 | 6 (11) | 49 (89) |
| 4-8 weeks | 0.53 [0.07, 3.87]; 0.535 | 0.52 [0.07, 3.75]; 0.514 | 1 (1) | 157 (99) | 1.08 [0.65, 1.79]; 0.779 | 1.08 [0.65, 1.80]; 0.766 | 17 (10) | 157 (90) |
| 8-12 weeks | 1.12 [0.35, 3.59]; 0.844 | 1.07 [0.34, 3.43]; 0.903 | 3 (1) | 236 (99) | 1.56 [1.08, 2.24]; 0.017 | 1.55 [1.08, 2.24]; 0.018 | 36 (13) | 236 (87) |
| 12-16 weeks | 0.52 [0.13, 2.14]; 0.363 | 0.50 [0.12, 2.07]; 0.339 | 2 (1) | 304 (99) | 0.93 [0.63, 1.37]; 0.708 | 0.94 [0.64, 1.39]; 0.759 | 30 (9) | 304 (91) |
| 16-20 weeks | 0.70 [0.22, 2.25]; 0.549 | 0.69 [0.21, 2.20]; 0.526 | 3 (1) | 335 (99) | 0.78 [0.52, 1.16]; 0.212 | 0.80 [0.54, 1.19]; 0.271 | 28 (8) | 335 (92) |
| 20-24 weeks | 1.25 [0.50, 3.10]; 0.629 | 1.19 [0.48, 2.95]; 0.711 | 5 (1) | 359 (99) | 1.10 [0.78, 1.54]; 0.583 | 1.11 [0.79, 1.55]; 0.561 | 41 (10) | 359 (90) |
| 24-28 weeks | 1.75 [0.80, 3.79]; 0.159 | 1.69 [0.78, 3.66]; 0.186 | 7 (2) | 383 (98) | 1.00 [0.71, 1.41]; 0.999 | 1.02 [0.72, 1.44]; 0.913 | 39 (9) | 383 (91) |
| 28-32 weeks | 1.33 [0.58, 3.06]; 0.498 | 1.26 [0.55, 2.90]; 0.582 | 6 (1) | 402 (99) | 1.41 [1.05, 1.88]; 0.022 | 1.41 [1.05, 1.89]; 0.024 | 55 (12) | 402 (88) |
| 32-37 weeks | NA | NA | NA | 551 (100) | 1.12 [0.85, 1.48]; 0.410 | 1.17 [0.89, 1.55]; 0.266 | 61 (10) | 551 (90) |

Numbers are odds ratios [95% confidence interval]; p-value, or N (%). Adjusted for gravidity; clinic site; yearly malaria incidence; and malaria history (within the current pregnancy) (see Figure S1). ^*^The reference group within each time interval is women with no malaria detected within each respective time interval. ^*^The reference group for malaria at time *t* is no malaria detected at time *t*; the reference group for a/symptomatic malaria at time *t* is no malaria (either asymptomatic or symptomatic) detected at time *t.*
