# Supplementary material for: Influence of the number and timing of malaria episodes during pregnancy on prematurity and small-for-gestational-age in an area of low transmission
Source: BMC Med. 2017 Jun 21;15:117. doi: 10.1186/s12916-017-0877-6 (PMC5479010; doi:10.1186/s12916-017-0877-6)
Supplement: Supplementary file 7 — Sub-group analyses to explore the influence of changes over time. (DOCX 322 kb) [file 12916_2017_877_MOESM7_ESM.docx]

**Additional file 7: Sub-group analyses to explore the influence of changes over time**

Data was collected over a 30-year period, and the influence of changes over time in malaria transmission, methods used to estimate gestational age, antimalarials, and the quality of antenatal and obstetric care on our results is unknown, and therefore limits the reliability of these results. We have performed sub-group analyses pre- and post-2001, which was when ultrasound was introduced to SMRU antenatal clinics.

**Figure 1. The association between the number of malaria episodes in pregnancy and small-for-gestational-age (SGA) – pre-2001 (left) and post-2001 (right).**

**Figure 2. The association between the gestational age at falciparum or vivax malaria detection and treatment and small-for-gestational-age (SGA) – pre-2001 (left) and post-2001 (right).**

**Figure 3. Pre-2001: The association between the gestational age at falciparum or vivax malaria detection and treatment and preterm birth – pre-2001 (left) and post-2001 (right).**
